# Candidate Gene Screen in the Red Flour Beetle *Tribolium* Reveals *Six3* as Ancient Regulator of Anterior Median Head and Central Complex Development

**DOI:** 10.1371/journal.pgen.1002416

**Published:** 2011-12-22

**Authors:** Nico Posnien, Nikolaus Dieter Bernhard Koniszewski, Hendrikje Jeannette Hein, Gregor Bucher

**Affiliations:** 1 Center for Molecular Physiology of the Brain (CMPB), Göttingen Center of Molecular Biology, Caspari-Haus, Georg-August-University Göttingen, Göttingen, Germany; 2 School of Life Sciences, Oxford Brookes University, Oxford, United Kingdom; 3 MPI für Molekulare Genetik, Berlin, Germany; New York University, United States of America

## Abstract

Several highly conserved genes play a role in anterior neural plate patterning of vertebrates and in head and brain patterning of insects. However, head involution in *Drosophila* has impeded a systematic identification of genes required for insect head formation. Therefore, we use the red flour beetle *Tribolium castaneum* in order to comprehensively test the function of orthologs of vertebrate neural plate patterning genes for a function in insect head development. RNAi analysis reveals that most of these genes are indeed required for insect head capsule patterning, and we also identified several genes that had not been implicated in this process before. Furthermore, we show that *Tc-six3/optix* acts upstream of *Tc-wingless*, *Tc-orthodenticle1*, and *Tc-eyeless* to control anterior median development. Finally, we demonstrate that *Tc-six3/optix* is the first gene known to be required for the embryonic formation of the central complex, a midline-spanning brain part connected to the neuroendocrine pars intercerebralis. These functions are very likely conserved among bilaterians since vertebrate *six3* is required for neuroendocrine and median brain development with certain mutations leading to holoprosencephaly.

## Author Summary

All bilaterian animals evolved from one common ancestor. Previous gene function analyses have revealed that several genes play a role in the patterning of anterior regions in all bilaterian animals, suggesting similar mechanisms underlying anterior nervous system formation in humans and the patterning of the insect head and brain. In order to identify novel genes required for anterior development in insects, we have systematically tested genes known to be crucially involved in early nervous system development in vertebrates (e.g. mice and humans) for their activity in the head of the red flour beetle *Tribolium casteneum*. Indeed, all but one of these genes are required for head development. Intriguingly, we find that *six3* is required for anterior median brain structures in insects just as it is in vertebrates, where *six3* mutations lead to holoprosencephaly.

## Introduction

The insect head is composed of several fused segments, the number of which remains disputed (e.g. [1]–[6]). The posterior labial, maxillary and mandibular segments are patterned by the well-studied trunk segmentation cascade. In contrast, the patterning of the procephalic region (intercalary, antennal, ocular segments and anterior non-segmental region) is less well understood. It has been suggested in the fruit fly *Drosophila melanogaster* that the head gap-like genes *orthodenticle (otd)*, *empty-spiracles (ems)*, *buttonhead (btd)* and *sloppy-paired (slp)* activate segment polarity genes directly or via second order regulators [7]–[9] but an instructive role could not be confirmed for *btd* and *ems* [10], [11]. Moreover, the segment polarity interactions of head segments differ from those in the trunk. For example, *hedgehog (hh)* expression in the intercalary segment is driven by its own unique enhancer element [12], [13] (see [4] and [6] for further details).

However, the development of the larval head of *Drosophila* is highly derived. During late stages of embryogenesis, the head gets turned outside into the thorax (head involution) and consequently cuticular head structures are highly reduced [14]–[16]. Also the emergence of the everted adult head from imaginal discs is derived within insects [17], [18]. These morphological differences correlate with changes in embryonic pattern formation. Comparisons with other insects have revealed that the upstream levels of head formation differ profoundly (e.g. no *bicoid* in most insects [19], no *torso* signaling in head development [20], different input of *decapentaplegic (dpp)* on head development [21]) while some degree of conservation is found on lower levels like the head gap like genes, second order regulators and segment polarity genes ([4] and references therein). Notably, the head expression of *wingless (wg)* appears to be reduced in *Drosophila* correlating with its derived head development. Hence, the evolution of the *Drosophila* head involved both structural changes and alterations of the gene regulatory network [4], [6], [14], [22].

Intriguingly, orthologs of several genes required for *Drosophila* head patterning play a role in vertebrate neural plate patterning *(e.g. otd/otx, ems/emx, slp/bf1, tailless(tll)/tlx)*. These data indicated that anterior patterning in insects and vertebrates relies on a strongly overlapping gene set [2], [7], [8], [13], [23]–[36]. Indeed, additional similarities between vertebrate and insect head and brain patterning have subsequently been revealed (e.g. [37]–[47]). Furthermore, an urbilaterian origin of anterior brain patterning has also been supported by similar data in an annelid [48]–[52].

The red flour beetle *Tribolium castaneum* has recently been established as a model for insect head development because it has an insect typical non-involuted head developing from a ventral-posterior region of the blastoderm, reflecting the ancestral situation (reviewed in [4], [53]). Several orthologs of *Drosophila* head patterning genes have been studied in *Tribolium* revealing differences with respect to the head gap-like genes [54] and *knirps* [55] but also a number of similarities with respect to second order regulators [56], [57]. Furthermore, genes required for vertebrate placode development were found to be active at similar locations in the *Tribolium* embryonic head [44].

*Tc-six3* is a member of the six type homeobox gene family, which has three members in insects [58] while two paralogs of each family are found in mouse *(six3/six6, six1/2 and six4/5)*. It is required for the formation of the labrum, an appendage of the non-segmental part of the head [3]. The *Drosophila* ortholog, called *optix*, is required for eye development [59]–[61], and for maxillary and clypeolabral structures of the larval head [62]. However, genetic interactions of this gene in the context of head development have not been analyzed in insects so far. Suggestively, the vertebrate *six3* gene is essential for eye development [63]–[70] and anterior neural plate patterning [71]–[75]. Furthermore, vertebrate *six3* and its paralog *six6* are involved in the development of the neuroendocrine pituitary and hypothalamus [76]–[80]. As the *six3* expression domain is anterior to the *otd* domain in arthropods, annelids and vertebrates, it is likely that this was also a feature of the last common ancestor of bilaterian animals [81].

In order to identify novel insect head patterning genes and with the high degree of conservation in mind, we comprehensively tested *Tribolium* orthologs of vertebrate neural plate patterning genes for a function in the insect head. Indeed, we find that many of them are required for head development. Closer examination of *Tc- six3* reveals that it acts upstream of *Tc-wingless (Tc-wg), Tc-otd and Tc-eyeless (Tc-ey)* in anterior median head patterning. Further, we find that *Tc-six3* is required for median brain development with a specific role in central body formation.

## Results

### 18 out of 24 Vertebrate Neural Plate Patterning Genes Are Expressed in the *Tribolium* Head

**Figure 1:**
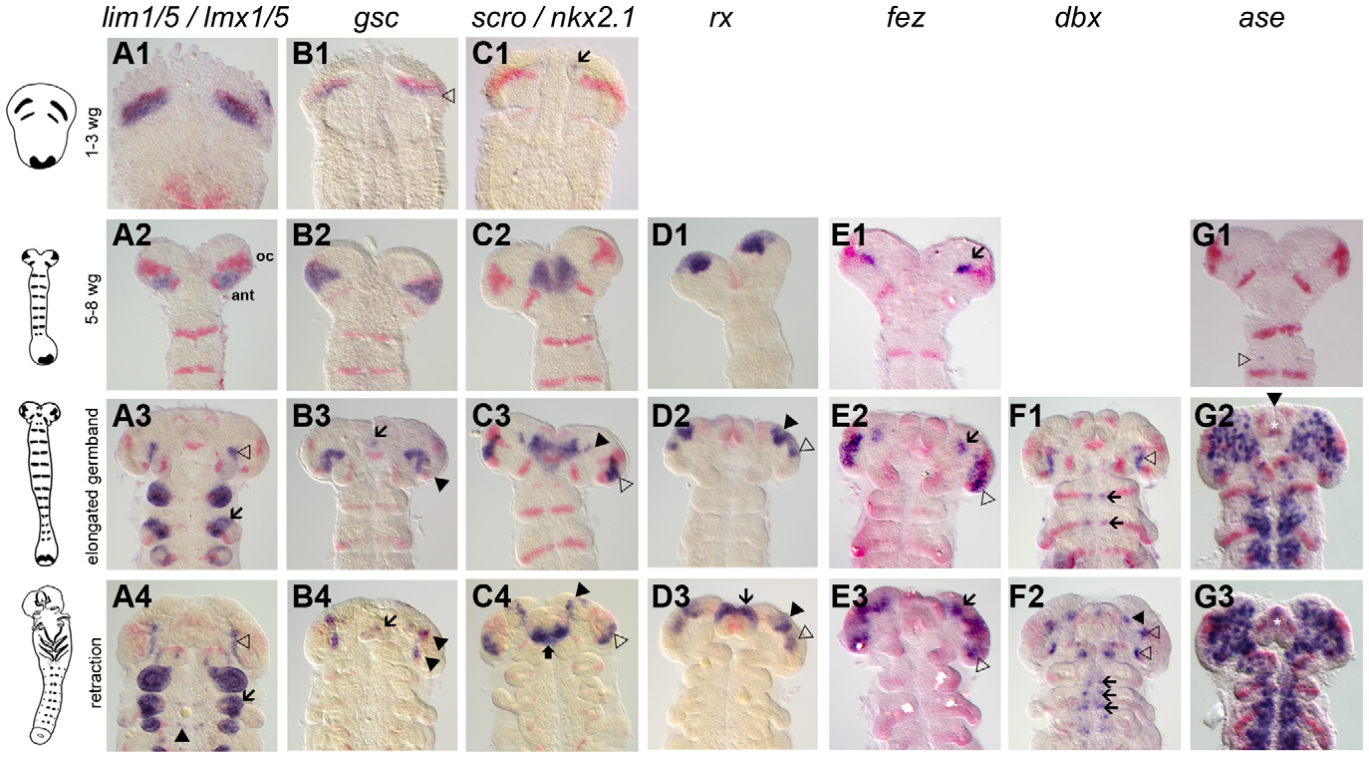
Novel genes with anterior but not segmental expression. Gene expression depicted in blue with *Tc-wg* as red counterstain. Germband stages are oriented with anterior to the top. The embryos in one row are staged according to their *Tc-wg* pattern and shape. Stages prior to onset of expression are omitted. (A) *Tc-lim1/5* expression starts in parallel to the ocular *Tc-wg* domain (A1) before it covers parts of the antennal segment (A2). Later, this domain shrinks (open arrowhead in A3) and expression in limb buds (arrow in A3–4) and in some cells in the CNS starts (black arrowhead in A4). (B) *Tc-gsc* expression starts in parallel to the ocular *Tc-wg* domain (open arrowhead in B1) before covering parts of the antennal segment (B2). Some cells close to the eye anlagen express *Tc-gsc* (black arrowheads in B3–4) and eventually stomodeal expression starts (arrow in B3–4). (C) *Tc-scro* expression starts as bilateral median dots (arrow in C1) and expands to cover an anterior median region with the stomodeum in the center (C2). Additional expression is found in the eye anlagen (open arrowhead in C3–4), in lateral/anterior cells (black arrowhead in C3–4) and in the labrum (black arrow in C4). (D) *Tc-rx* expression starts in elongating embryos in two bilateral patches, which are later subdivided (black and open arrowheads in D2–3). Still later, an expression at the base of the labrum arises (arrow in D3). (E) Initial *Tc-fez* expression covers a very restricted domain (arrow in E1–3). Later, an additional domain in the eye anlagen develops (open arrowheads in E2–3). (F) In addition to segmental dots (arrows in F1–2), strong *Tc-dbx* expression is found close to the ocular *Tc-wg* domain (open arrowhead in F1) which later splits (open arrowheads in F2). A more anterior domain becomes visible at later stages (black arrowhead in F2). (G) *Tc-ase* is used as marker for neuroectoderm because it is expressed in recently delaminated neural stem cells (neuroblasts). Expression starts in the 5–8 *wg*-stripe stage embryo (open arrowhead in G1). The neuroectoderm consists of a salt and pepper pattern of neural and epidermal precursors (G2–3). Note the stomodeal neuroblasts (white star in G2–3) in an otherwise non-neural anterior median region.

From the literature we identified 24 genes involved in early vertebrate neural plate patterning (see Table S1) [32], [33], [41], [45], [74], [80], [82]–[127]. Three genes do not possess orthologs in either the *Tribolium* or *Drosophila* genome (*Dmbx1/Atx*, *Vax1*, *Hesx1/Rpx*; see phylogenetic trees in Figure S1). Tc-FGF8 does not cluster unequivocally with mouse FGF8 but with the *Drosophila* Pyramus and Thisbe proteins which have previously been identified as FGF8 orthologs [128], [129]. Of the 21 orthologs, *Tc-BarH*, *Tc-Wnt11* and *Tc-munster/arx* are not expressed in the head anlagen (not shown), while the remaining 18 genes are active in the embryonic head. For these genes, we determined the expression pattern at several stages (determined by *Tc-wg* counter stain) in order to reveal their dynamics during head development. Genes that had not been described before in *Tribolium* are shown in Figure 1. In order to produce a comprehensive dataset with comparable staging, we also included previously described genes (Figures S2 and S3) (*Tc-otd* [130], *Tc-ems* [54], *Tc-tailless (Tc-tll)* [131], *Tc-six3* [3], *Tc-hedgehog (Tc-hh)* [132], [133], *Tc-cubitus-interruptus (Tc-ci)* [132], *Tc-wg* [134], *Tc-fgf8* [128], *Tc-sloppy paired (Tc-slp)* [135], *Tc-eyeless (Tc-ey)* [136], *Tc-ptx* [44], *Tc-irx* [137]).

Interestingly, a number of these genes are expressed in the head but not segmentally reiterated in the trunk *(Tc-otd, Tc-six3, Tc-tll, Tc-lim1, Tc-gsc, Tc-scro, Tc-rx, Tc-fez1)* supporting the notion that the anterior patterning system differs from the one of the trunk. However, other genes do have segmentally reiterated expression in addition to anterior expression *(Tc-hh, Tc-wg, Tc-ci, Tc-irx/mirr, Tc-fgf8, Tc-slp, Tc-ems, Tc-ey, Tc-dbx, Tc-ptx)*, linking these two systems.

### All Genes But One Are Involved in Head Epidermis Patterning

**Figure 2:**
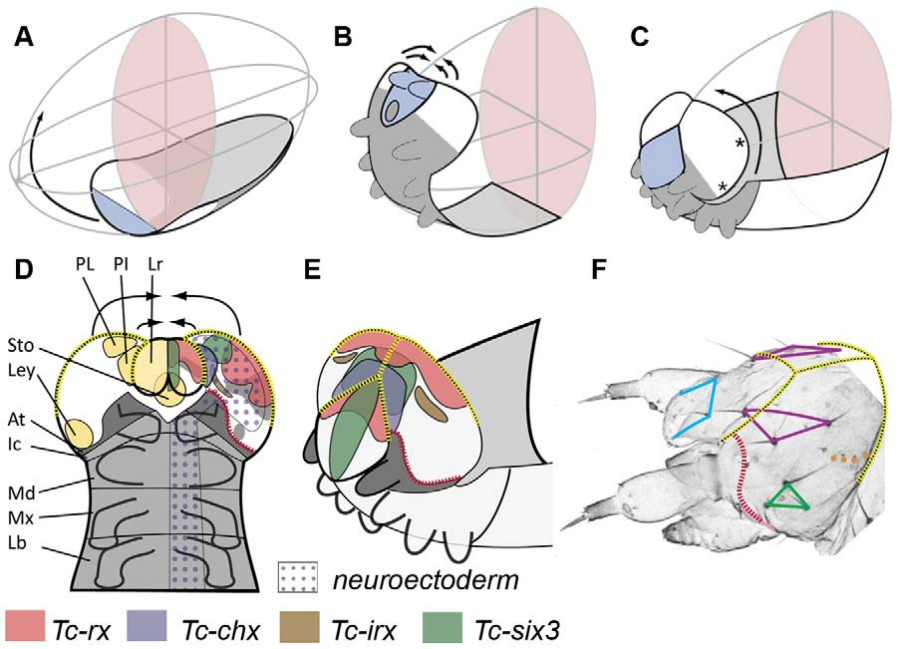
Fate map and morphogenesis of the head. (A–C) Our recently proposed “bend and zipper” model of head development describes the movement of the ventral head anlagen (A) towards the anterior, their upward bending (B) and their zippering together at the dorsal midline (C) (after [137]). Hence, the anterior lateral tissue (white in A–C) forms the dorsal and lateral head capsule. Many of the genes shown in Figure 1 and Figures S2 and S3 are expressed in the preantennal region, hence, cuticle phenotypes are expected to affect the head capsule (see text for further details). (D) Schematic of a retracting germband embryo with segments shown in grey (At = antennal (dark gray); Ic = intercalary; Md = mandibular; Mx = maxillary; Lb = labial). Light yellow shaded domains in the left head lobe indicate the approximate location of the neuroendocrine anlagen pars lateralis (PL, marked by *Tc-fas2*) and pars intercerebralis (PI, marked by *Tc-chx*; both markers established in *Drosophila* by [141]). Further, the stomodeum with stomatogastric nervous system anlagen (Sto), and the larval eye anlagen (Ley, based on [136]) are shown. The neuroectoderm is marked by the dotted field in the right half of the embryo (based on the neuroblast marker *Tc-asense*). Expression patterns of head markers in flat preparations of retracting embryos are indicated in different colors in the right head lobe (compare to Figure 1 and Figures S2 and S3). (E) Expression of the same genes is shown in a non-flattened embryo, in which the head is zippering together at the dorsal midline (based on Figure S6). (F) Head cuticle of a first instar larva with the location of the most prominent head bristles marked by the corners of the colored shapes (see Figure S4 for complete pattern). The yellow broken lines in D–F help to relate the tissue boundaries of a retracting embryo with the L1 head. The red broken line indicates the adjacent expression of ocular *Tc-hh* and *Tc-wg* during early embryogenesis (see Figure S3A) and by *Tc-tll* and *Tc-ems* at later stages (see Figure S2 and S3). This fate map remains an approximation because it assumes that cells continue to express a certain gene and that the relative locations of cell groups do not change during the development from retracted germband stage to the hatchling.

The embryonic preantennal region gives rise to the lateral and dorsal head capsule (compare white area anterior to the dark grey shaded antennal segment in a flattened germband in Figure 2D with a non-flattened embryo depicted in E) [4], [44]. This region is marked by an invariant bristle pattern in the first instar larval cuticle [54] (see Figure 2F for most prominent bristles and Figure S4 for more details), which allows the localization of cuticle defects, which arose in pre-antennal tissues. Unfortunately, previously published RNAi phenotypes had not been scored for the head bristle pattern except for *Tc-otd* and *Tc-ems* [54] and *Tc-ey/toy*, where a small subset of three setae was scored [136]. Therefore, we performed RNAi for all novel genes as well for those where the head capsule defects had not been described previously. We excluded *Tc-hh* and *Tc-wg* because RNAi for these genes induces severe generalized embryonic defects, which impede the analysis of the bristle pattern (data not shown and [132], [138]).

**Figure 3:**
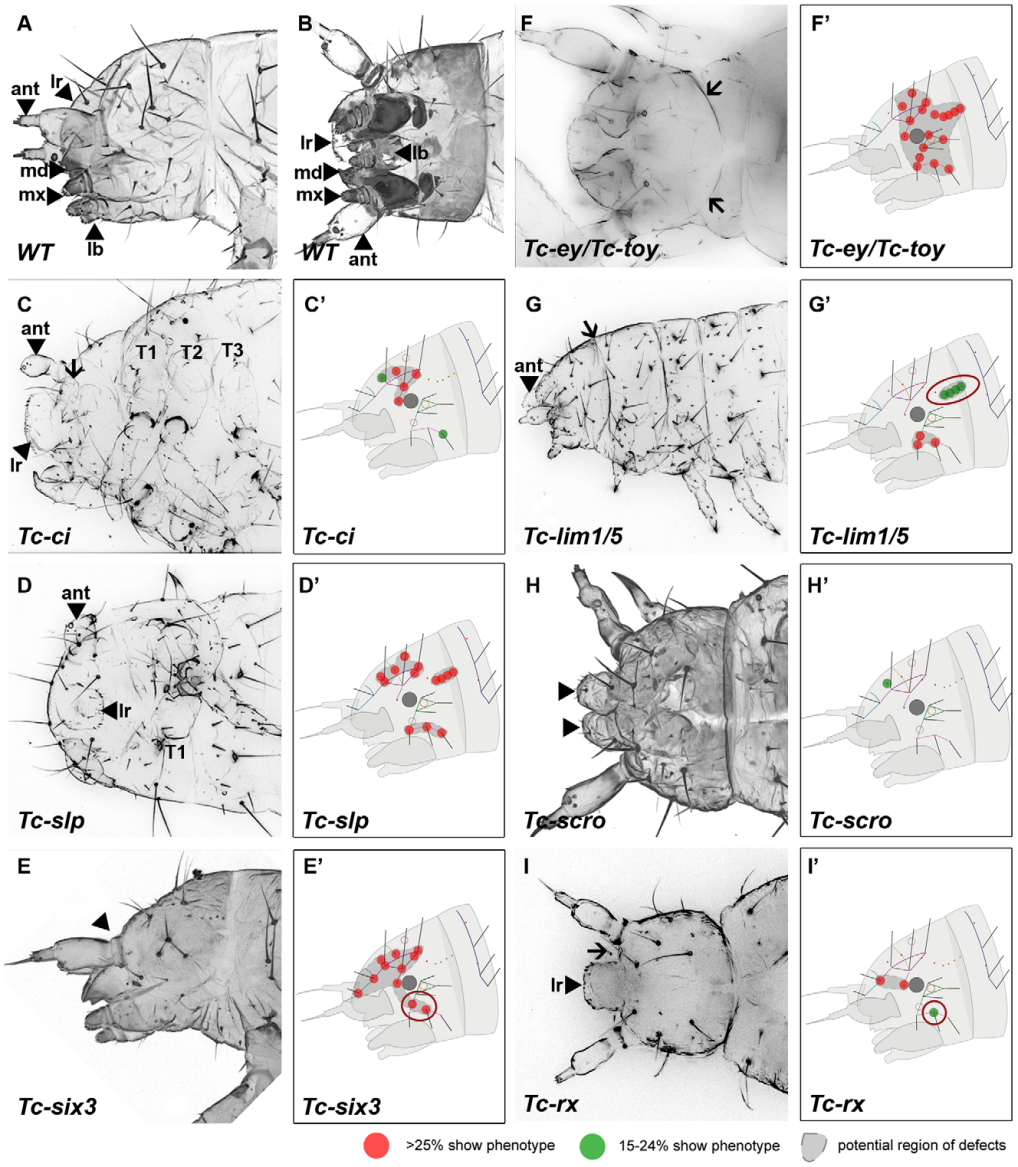
Genes whose knock down result in severe defects in larval cuticles. Larval head cuticles and schematic representations of respective head bristle pattern defects. All larval heads are oriented with the anterior to the left. See Figure 2D–2F for fate map. The wildtype bristle pattern is shown in Figure S4 (based on Schinko et al. 2008 but extended with the dorsal ridge row). Red dots indicate disturbance in >25%, green dots in 15–24% of scored cuticles (see Table S2 for quantification). Assuming that tissue surrounding a given bristle is affected as well, a putative region of defects is marked in dark grey (C′–I′). The location of the cuticle defects generally correspond to the embryonic expression domains of the respective genes – exceptions are highlighted with red circles (G′, E′, I′). (A–B) Lateral and ventral view of a wild type larval head cuticle with head bristle pattern, labrum (lr), antenna (ant), mandibles (md), maxillae (mx) and labium (lb). (C) *Tc-ci* RNAi affects gnathal segments and antennae probably due to its role in segmental Hedgehog signaling. In addition, some dorsal head bristles are strongly affected. (D) *Tc-slp* RNAi strongly affects gnathal segments in line with being a pair rule gene. Widespread dorsal bristle defects confirm the function in anterior patterning. (E) *Tc-six3* RNAi leads to loss of the labrum (black arrowhead in E) and adjacent head bristles. (F) *Tc-ey/toy* double RNAi leads to strong reduction of head size and disturbance of the “neck” (arrows). In addition, almost all head bristles are strongly affected (see Figure 4 for respective single RNAi). (G) Gnathal appendages are malformed in *Tc-lim1/5* RNAi, consistent with its expression in that domain. Strong defects in the lateral head and overall shortening of the head capsule are observed. (H) The labrum is not fused in *Tc-scro* RNAi embryos (black arrowheads) and one head bristle is affected to some extent. (I) *Tc-rx* RNAi results in reduction of the labrum and an increased distance between the labrum and the antennae (arrow) while two nearby bristles are affected frequently.

*Tc-ci* RNAi interfered with segmentation of the entire embryo as described [132]. Head defects ranged from the total loss of the head (9.1%, n = 11) to the loss and malformation of gnathal segments (90.9%; Figure 3C). Where accessible, the head bristle pattern was analyzed, revealing mainly a disruption of the vertex setae marking the dorsal tissue (Figure 3C′; the numbers for this and other bristle pattern defects are given in Table S2, the names of the setae and bristles are given in Figure S4). Knock down of the pair rule gene *Tc-slp* resulted in a pair rule phenotype [135] (70%, n = 10; Figure 3D). We found additional head defects in the median part of the vertex, the bell row and the maxilla escort bristles (Figure 3D′). *Tc-six3* knock down leads to loss of the labrum and clypeolabral parts of the anterior head capsule [3], [62] (100%, n = 16; Figure 3E). In line with the loss of anterior median cuticle, the anterior vertex seta and the anterior vertex bristle were missing (Figure 3E′), while the median part of the dorsal head cuticle often displayed an irregular pattern of additional bristles and setae. *Tc-ey and Tc-toy* have been shown to act synergistically in eye formation, while the respective analysis on the head bristle pattern was restricted to 3 bristles [136]. Re-analysis of single and double RNAi revealed more extensive defects than previously described (Figure 3F, 3F′). In comparison with single RNAi experiments, double RNAi revealed a 1,4- to 6-fold increase of penetrance of bristle pattern defects using the same final concentration of dsRNA (Table S2) confirming that the two *Tribolium pax6* orthologs also act synergistically in epidermis development. The head of *Tc-lim1/5* RNAi larvae was compacted and shortened (16.7%, n = 12; Figure 3G). Head appendages were present but mostly malformed (41.7%). The anterior and median maxilla escort bristles failed to form (Figure 3G′), while in 20.8% no bell row was observed (Figure 3G′). In *Tc-scro* RNAi larvae the labrum failed to fuse either completely (60%; n = 15) (black arrowheads in Figure 3H) or partially (13.3%; not shown). The bristle pattern remained largely unaltered except for the anterior vertex bristle (Figure 3H′). Interestingly, the labrum quartet bristles were present even on unfused labra. In *Tc-rx* knock down larvae, the labrum was narrower than in wild type leading to a widened space between the labrum and the antennae (25%, n = 8; arrow in Figure 3I) and the adjacent clypeus bristles of the labrum quartet were lost in more than half of the analyzed RNAi larvae (Figure 3I′). Additionally, the antenna basis bristle and the median maxilla escort seta were sensitive to *Tc-rx* knock down (Figure 3I′).

**Figure 4:**
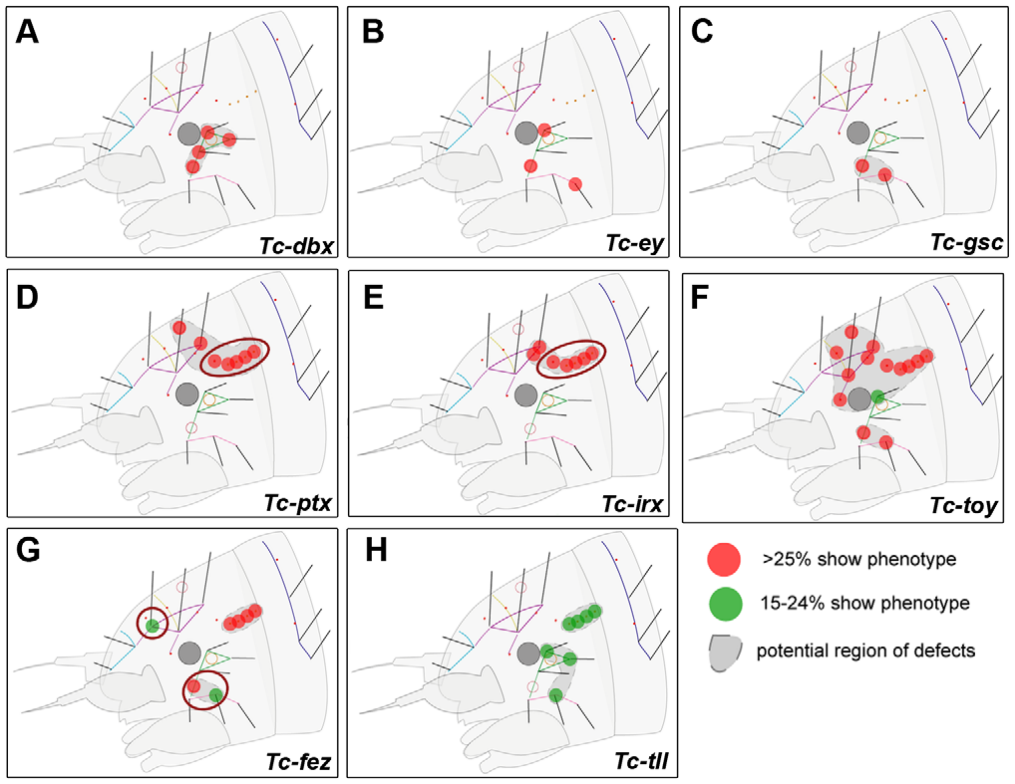
Genes whose knock down results in minor defects in larval cuticles. RNAi against these genes did not lead to strong head capsule or head appendage defects (not shown). Hence, only schematic representations of bristle pattern defects are depicted (see Table S2 for quantification). Cuticle defects, which do not correspond to the embryonic expression domains of the respective genes are marked by red circles (D, E, G). (A–C) The knock down of *Tc-dbx*, *Tc-ey/pax6* or *Tc-gsc* affects the formation of lateral bristles. (D–E) More dorsal defects are found after RNAi against *Tc-ptx* and *Tc- irx*. (F) *Tc-toy* single RNAi leads to widespread disturbance of head bristles while the paralog *Tc-ey* has a more restricted effect (compare with B). Only double RNAi leads to strong head capsule defects, indicating a synergistic function (Figure 3F). (G–H) *Tc-fez* and *Tc-tll* RNAi results in rather widespread defects.

RNAi against the remaining genes did not elicit large deletions but alterations of the head bristle pattern (Figure 4 and Table S2). Lateral defects were found after RNAi against *Tc-dbx*, *Tc-ey* single RNAi and *Tc-gsc* (Figure 4A–4C). *Tc-ptx* and *Tc-irx* led to dorsal defects (Figure 4D, 4E) while the bristle defects of *Tc-toy*, *Tc-fez* and *Tc-tll* were more widespread (Figure 4F–4H). No bristles were missing in *Tc-fgf8* RNAi (n = 29), although lethality of most larvae and a bent flagellum phenotype of the antenna in 41.5% (n = 53, not shown) confirmed the RNAi effect. In summary, we showed that all vertebrate neural plate patterning genes investigated here (except for *Tc-fgf8*) are indeed involved in anterior head epidermis patterning in *Tribolium*. By and large, the cuticle defects correspond well with the location of the expression domain, but we also find some indication for indirect defects (see red circles in Figure 3 and Figure 4 and discussion for details).

### *Tc-six3* Is a Repressor of *Tc-wg* and *Tc-otd1*

**Figure 5:**
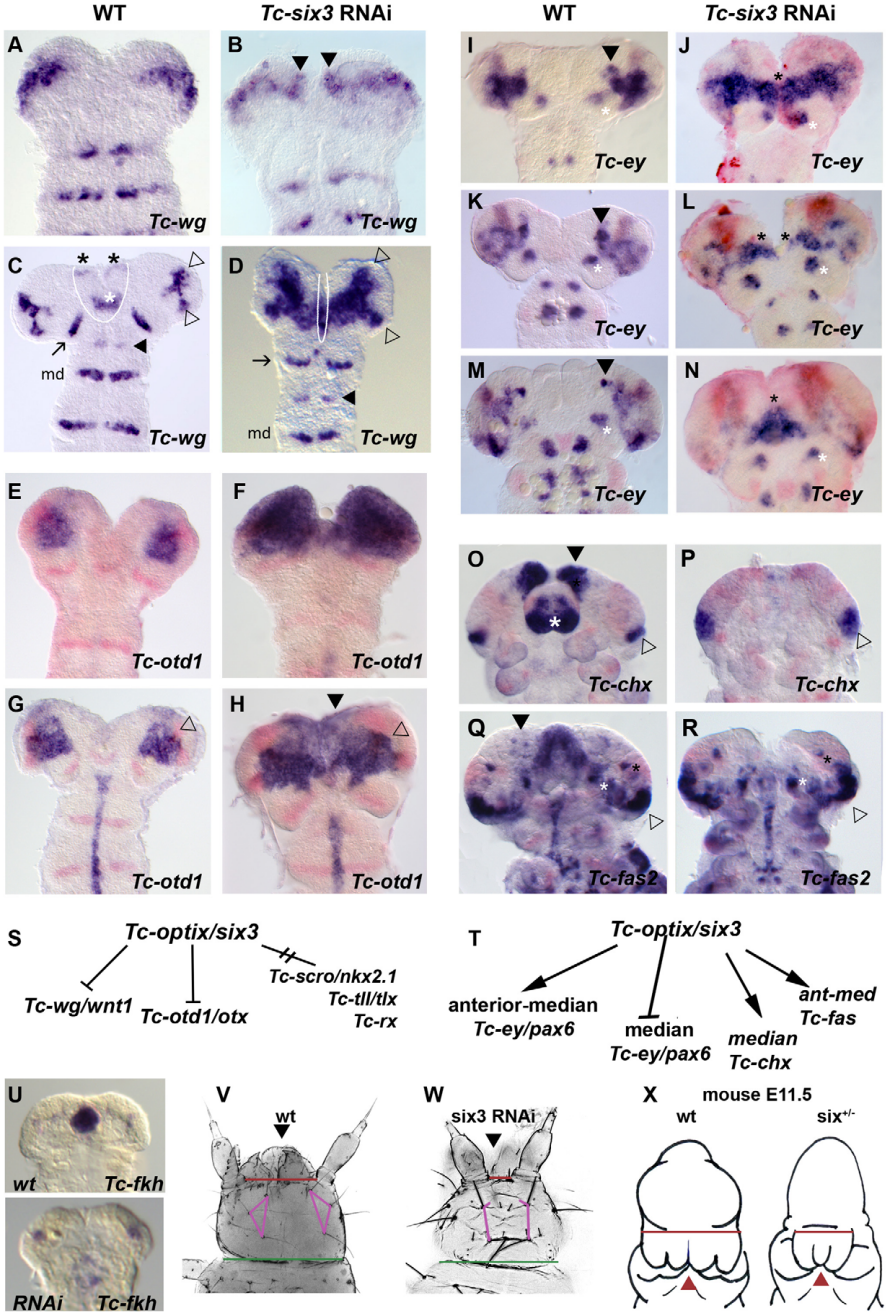
*Tc-six3* is an upstream regulator of the anterior median region of the embryo. Heads are oriented with anterior to the top. *Tc-wg* expression is shown in red in E–R. (A–D) Upon *Tc-six3* RNAi, *Tc-wg* is derepressed in the median head (black arrowheads in B, strong medial domain in D) while the lateral parts appear unchanged (compare open arrowheads in D with C). Moreover, an anterior median region including the labrum and stomodeum (black and white asterisks in C, respectively) is lost (compare region outlined by white line in C with D). Intriguingly, the loss of median tissue leads to antennal *Tc-wg* stripes oriented perpendicular to the body axis (like all trunk stripes) in RNAi embryos. In wildtype, in contrast, they are turned outwards by the intervening tissue (compare distance and orientation of antennal stripes marked by arrow in D with C). Antennal, intercalary and mandibular domains are marked with black arrow, black arrowhead and md in both panels, respectively. (E–H) Upon *Tc-six3* RNAi, *Tc-otd1* is derepressed medially and anteriorly (e.g. black arrowhead in H) while the lateral expression appears normal (open arrowhead in G and H). (I–N) The effect of *Tc-six3* RNAi is different with respect to the various domains of *Tc-ey*. The major ocular domain is expanded medially (J) and ectopic medial expression can remain paired (black stars in L) or fused at the midline (black star in N). The mushroom body domain of *Tc-ey* (black arrowhead in I,K,M) is lost in *Tc-six3* RNAi embryos while the antennal domains are not altered (white asterisks in I–N). (O–R) *Tc-six3* RNAi leads to loss of neuroendocrine markers. (O–P) The pars intercerebralis anlagen marked by *Tc-chx* (black arrowhead) and the labrum (white asterisk) are lost in *Tc-six3* RNAi embryos while the *Tc-chx* eye domain remains present (open arrowhead in O,P). (Q–R) *Tc-fas2* marks neuroendocrine cells of the pars lateralis (black arrowhead in Q), which are lost upon *Tc-six3* RNAi, while more lateral aspects including eye expression remain unchanged (black and white stars and open arrowhead). (S–T) Summary of genetic interactions of *Tc-six3* identified in this work. *Tc-scro*, *Tc-tll* and *Tc-rx* are not altered after *Tc-six3* RNAi (not shown). (U–W) *Tc-six3* RNAi leads to the loss of anterior median structures like the stomodeum as shown by the loss of *Tc-fkh* expression (lower panel of U). (V–W) In dorsal views of head cuticles, loss of the labrum (black arrowhead) and the anterior vertex bristle, and a reduced distance between the antennae (red bar) and the head bristles (pink lines) are evident while more posterior aspects like the width of the neck (green bar) remain unchanged. (X) Strikingly, loss of anterior median head and brain tissue (holoprosencephaly phenotype) is also found in hypomorphic *six3* mutants in mouse embryos, where the distance between the eyes is reduced (red bar) and median structures are lost or fused (red arrowhead). Embryos are redrawn from Geng et al. 2008 [170].

Considering its early expression and severe RNAi phenotype, *Tc-six3* was likely to play a central role in insect head patterning. Therefore, we centered our subsequent analysis on the epidermal and neural function of this gene. We tested the effect of *Tc-six3* RNAi on genes that-based on our expression and RNAi data-were likely to interact. Indeed, the protocerebral neuroectodermal expression domain of *Tc-wg* (pne) [133] (also called the ocular *Tc-wg* domain or the head blob in *Drosophila* [2], [133]) expanded medially and anteriorly in early elongating RNAi embryos (black arrowheads in Figure 5B), resulting in massive median misexpression in fully elongated germbands (Figure 5D). The lateral aspects of *Tc-wg* expression appeared largely unchanged (open arrows in Figure 5C–5D). Moreover, loss of median embryonic tissue was apparent (white outline in Figure 5C–5D) including the stomodeum (see also Figure 5U) and labrum anlagen (white and black stars in Figure 5C, respectively). As a consequence, the head lobes were not bent outwards and the antennal *Tc-wg* stripes became perpendicular to the body axis instead of being twisted outwards as in wildtype (compare arrows in Figure 5D with 5C; see Figure S5L–S50 for phenotypes of more advanced stages where the assignment of the antennal stripe becomes obvious). The expression of *Tc-otd1* was strongly expanded towards anterior and median tissue (compare Figure 5F, 5H with Figure 5E, 5G) while the lateral aspects appeared normal (open arrowheads in Figure 5G–5H). Despite being partially coexpressed (*Tc-tll* and *Tc-scro*) or expressed adjacent to the *Tc-six3* domain (*Tc-rx*), the expression domains of these genes remained unchanged after *Tc- six3* RNAi (not shown).

### Early Ocular *Tc-ey/Pax6* Is Repressed by *Tc-six3* While More Anterior Expression Domains Require *Tc-six3* Function

The effect of *Tc-six3* RNAi was different with respect to the various domains of *Tc-ey* (Figure 5I–5N). *Tc-ey* expression starts in a prominent ocular domain (open arrowheads in Figure S3 H2–3) before an additional anterior median expression arises (black arrowheads in Figure 5I, 5K, 5M and Figure S3 H4–5). We found coexpression of *Tc-dachshund* with parts of the anterior median domain (white arrowheads in Figure S5C–S5C″), making it possible that it marks mushroom body anlagen as in *Drosophila* [139], [140]. In *Tc-six3* RNAi embryos, the early ocular domain was strongly expanded towards the midline (compare Figure 5J with Figure 5I). Later, a domain remained visible at the midline (black star in Figure 5N). The anterior median domain did not develop in *Tc-six3* RNAi embryos (Figure 5J, 5L, 5N). Again, the lateral aspects of the ocular domain as well as the segmental domains appeared unaffected.

### *Tc-six3* Is Essential for the Expression of Neuroendocrine Marker Genes

*six3* has been implicated in neuroendocrine development in vertebrates [80] and protostomes [81]; and in *Drosophila*, it is coexpressed with the neuroendocrine markers *fas2* and *chx* [81], [141]. The functional relevance of this co-expression remained unknown. We confirmed co-expression in *Tribolium* (Figure S5D–S5G, S5H–S5K) and found that in *Tc-six3* RNAi embryos the anterior median domains of *Tc-fas2* and *Tc-chx* were absent (compare domains marked by black arrowheads in Figure 5O, 5Q to Figure 5P, 5R).

### *Tc-six3* Is Required for Development of the Central Body and the Median Brain

**Figure 6:**
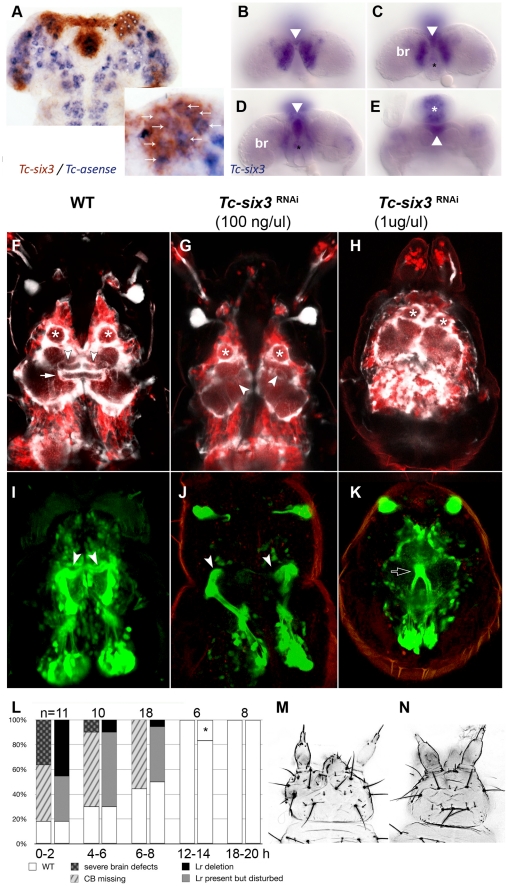
*Tc-six3* is required for central body development. (A) Fully elongated embryo stained for the neuroblast marker *Tc-asense* (blue) and *Tc-six3* (red). Eight neuroblasts arise from the lateral *Tc-six3* expression domain by 24 hours of development. (B–E) Staining of *Tc-six3* in 42–48 hour old embryos shown in different optical sections taken from dorsal. Expression is observed in the dorsal epidermis (B), in the median brain (br) lateral to the stomodeum (C), the stomodeal roof (D) and the labrum (white asterisk in E). The stomodeum is marked by a black asterisk, the labrum with a white asterisk. Note additional staining of *Tc-six3* in median cells (white arrowheads in B–E). (F–K) First instar larval brains are imaged from dorsal within the head cuticle. Anterior is up in all panels; white asterisks mark antennal lobes. (F,I) Wildtype brain with neural cells marked in red, glia shown in white (F) and mushroom bodies marked in green (I). The central body is a neuropil ensheathed with glia, which spans the midline (white arrow). It is located posterior to the median lobes of the mushroom bodies (white arrowheads in F,I). (G,J) After *Tc-six3* RNAi with low doses, the central body is not detectable in an otherwise normal brain. The mushroom bodies are intact but their median lobes do not meet at the midline (white arrowheads in G,J). (H,K) In strong RNAi phenotypes, the separation of the brain hemispheres is lost (H) and the mushroom bodies come into close apposition and are reduced in size (black arrow in K). (L) The strength of brain and epidermal phenotypes correlates. Injection of dsRNA against *Tc-six3* at different points in time after egg deposition lead to decreasing strength of the cuticle phenotype (right bars) and concomitant decrease of brain phenotype strength (left bars). The black star indicates that in one embryo, a strong cuticle defect was observed, which was not part of the phenotypic series of *Tc-six3*. As this was likely a background effect or injection artifact, this embryo was not considered as *Tc-six3* phenotype. (M) Weak cuticle phenotype with labrum reduced but present. (N) Strong phenotype – note the loss of the labrum and the reduced distance between the antennae.

In insects, epidermal and neural precursor cells are intermingled in the neuroectoderm. The neural stem cells receive spatial patterning cues before they delaminate from the neuroectoderm and contribute to the central nervous system in a cell autonomous way. The remaining epidermal cells eventually secrete the cuticle [142], [143]. This explains why mutations in segment polarity genes elicit both cuticular and CNS defects. With this in mind, it was likely that *Tc-six3* knock down would induce brain defects. First, we determined that eight *Tc-ase* marked neuroblasts are found within the *Tc-six3* marked neuroectoderm until 24 hours of development (extended germband stage, white stars and arrows in Figure 6A). Later, in 42–48 hour old embryos, *Tc-six3* is expressed in the developing brain lateral and anterior to the stomodeum (Figure 6C, stomodeum marked by black asterisk). Additionally, staining is evident in the overlaying dorsal epidermis (Figure 6B), the stomodeal roof (Figure 6D) and the labrum (Figure 6E).

In order to test the hypothesis of a neural function of *Tc-six3*, we generated transgenic imaging lines marking neural cells, glia and mushroom bodies (Koniszewski, Kollmann, Averof, in preparation) and we identified the central body at the L1 larval stage (white arrow in Figure 6F). Indeed, *Tc-six3* RNAi at low doses led to the loss of the central body in an otherwise normal brain (Figure 6G). Higher doses additionally reduced the distance between the two brain hemispheres (Figure 6H). In weak phenotypes the orientation of the median lobes of the mushroom bodies towards the midline was lost (compare white arrowheads in Figure 6J with Figure 6I) while in strong phenotypes, upon convergence of the brain hemispheres, the mushroom bodies approached each other and were reduced in size (see black arrow in Figure 6K).

In the light of the *Tc-six3* expression profile, these brain defects could be due to an early neuroectodermal function of *Tc-six3* (see neuroectodermal expression in Figure S2B) or to a later function in developing neural cells (see expression in the brain in Figure 6B–6E). In the first case, epidermal and neural phenotypes would be elicited at the same time and, hence, be tightly linked. In the second case, knockdown at late embryonic stages (when epidermal patterning is already finished) would lead to the induction of neural phenotypes in otherwise unaffected heads. To test this, we injected 1 ug/ul *Tc-six3* dsRNA in embryos at 0–2, 4–6, 6–8, 12–14 and 18–20 hours post egg laying and scored the resulting L1 larvae for both cuticle and brain phenotypes (Figure 6L). Indeed, the severity of neural and epidermal phenotypes correlated strongly and we never observed brain phenotypes in embryos without cuticle defects. This indicates that both cuticle and brain phenotype are outcomes of the same early neuroectodermal patterning events.

## Discussion

### Identification of Novel Insect Head Patterning Genes

With our candidate gene approach we identified five genes that had not been implicated in insect head epidermis patterning before *(goosecoid, scarecrow, fez1, dbx, ptx)*. For four additional genes, we show involvement in embryonic head capsule development while a role in adult *Drosophila* head patterning had been described previously (*ci*, *Drx*, *lim1*, *mirror*) [144]–[147]. Based on our fate map, the cuticle defects generally correspond well with the head expression of the respective gene. However, the bell row and the setae of the maxilla escort appear to be sensitive to indirect effects because they were affected in several RNAi experiments with genes, which-based on our fatemap-do not show expression in the respective regions (red circles in Figure 3 *Tc-lim1/5*, *Tc-ptx*, *Tc-irx* and Figure 4 *Tc-six3*, *Tc-rx*, *Tc-fez*). Both regions are located where the head lobes are predicted to fuse either with gnathal segments (maxilla escort) or the trunk (bell row; see black stars in Figure 2C). Hence, primary defects of a gene knockdown in head lobe morphology or size could lead to the observed secondary defects.

### A Novel Regulator of Central Complex Formation

We show that *Tc-six3* is required for proper formation of the central body, which is a midline spanning neuropile and part of the central complex. To our knowledge, this is the first gene known to be required for embryonic central complex development. Our data are in line with cell lineage tracing experiments in grasshopper embryos, where neuroblasts at a corresponding anterior median position contribute to the central complex [148]. Further, expression of *optix/six3* in corresponding neuroblasts was also shown in *Drosophila* [81]. Together, these data are consistent with the hypothesis that *Tc-six3* is required in the neuroectoderm for specifying the identity of central body neuroblasts. However, tools to genetically trace the offspring of these neuroblasts [149] are needed to prove this link.

In hemimetabolous insects, which represent the ancestral mode of embryogenesis, all neuropils of the central complex are formed during embryogenesis. In *Drosophila*, in contrast, the central complex develops during late larval stages [150]–[153]. *Tribolium* takes an intermediate position by forming a subset of central complex neuropils during embryogenesis, a situation apparently conserved with another tenebrionid beetle [154]. With the newly available brain imaging lines and its amenability to functional genomics *Tribolium* is an excellent model to investigate the genetics of embryonic central complex development.

### Anterior-Median Development in Insects

We showed that *Tc-six3* is expressed in an anterior median domain from earliest stages on and that it acts as an upstream component of anterior median patterning. *Drosophila optix/six3* is expressed in an anterior blastodermal ring anterior to *otd*, which persists at the dorsal side [58], [70], [81] and is required for labral and maxillary structures [62]. Its ring like expression does not support an involvement in median patterning but relevant genetic interactions remain to be studied. The later expression in the labrum and in bilateral dorsal domains, however, is similar in both species.

Interestingly, aspects of dorsal median head patterning are controlled by *dpp* in *Drosophila*. Shortly before gastrulation, the action of *dpp* and its downstream target *zen* at the dorsal midline separate the neuroectoderm into paired anlagen by medial repression of genes and by promoting median cell death. This results in the establishment of bilateral expression of marker genes of the respective brain parts (e.g. *Dchx* (pars intercerebralis); Fas2 and *Drx* (pars lateralis); *sine oculis* and *eyes-absent* (visual system)) [46], [141], [155], [156]. Actually, many other anterior patterning genes initiate their expression as unpaired domains across the dorsal midline that are subsequently medially subdivided in *Drosophila* (e.g. *otd* [157], *tll* [158], *fezf* [159], *Dsix4* [58]). In contrast, the *Tribolium* orthologs of most of these genes are initiated as separate bilateral domains (*Tc-rx* and *Tc-fez* (Figure 1E and 1D), *Tc-c*hx and *Tc-Fas2* (Figure S5), *Tc-tll* [131]), *Tc-six4* [44], *Tc-sine oculis*, *Tc-eyes-absent* [160]). *Tc-otd1* starts out with ubiquitous expression related to axis formation [130], [161], [162] but then resolves into paired head lobe domains which are separate as with the aforementioned genes.

Due to differences in topology of the head anlagen (see below), median repression of anterior patterning genes by *Tc-dpp* is not required in *Tribolium*. Nevertheless, it is expressed along the rim of the head anlagen at blastoderm stages, some parts of which will become the site of dorsal fusion [163], [164]. However, Tc-Dpp activity (detected by antibodies against pMad) does not occur at the site of expression and is clearly distant from the arising *Tc-rx*, *Tc-chx*, *Tc-six4*, *Tc-sine oculis* or *Tc-fas2* domains [21]. Also the *Tc-dpp* RNAi phenotypes differ from *Drosophila* mutants in that the head anlagen are expanded and appear to have lost their dorso-ventral orientation (shown by expansion of *Tc-otd1* and the proneural gene *Tc-ASH*) in an overall ventralized embryo [21]. Hence, the early expression of *dpp* at the future dorsal midline might be ancestral, but its function with respect to medially repressing gene expression has probably evolved in *Drosophila*.

### *Tribolium* Probably Reflects the More Ancestral Situation

The difference in generation of paired dorsal domains in these two insect species reflects the different location of the head anlagen. In the long germ insect *Drosophila*, extraembryonic tissues are reduced to the dorsally located amnioserosa while the head anlagen are situated in the anterior dorsal blastoderm from earliest stages on [165], [166]. Consequently, the head lobes are never separated along the midline. In contrast, in the short germ insect *Tribolium*, the anterior blastoderm gives rise to extraembryonic amnion and serosa, which eventually ensheath the embryo [166]. In contrast to *Drosophila*, the *Tribolium* head anlagen are located in the ventral median blastoderm from where they move towards anteriorly and bend dorsally. The head lobes are separate from the beginning but fuse at late stages at the dorsal midline forming the dorsal head (bend and zipper model, see Figure 2A–2C and [4], [137] for more details). During these morphogenetic movements, the initially separate expression domains of the head lobes eventually come into close proximity at the dorsal midline like in *Drosophila* (Figure 2D–2F). Both the anterior dorsal location of extraembryonic tissue anlagen and the ventral location of the head anlagen are found in most insects [166] and in the hemimetabolous milkweed bug *Oncopeltus fasciatus*, gene expression data show a clear separated origin of the head lobes in the blastoderm [167]. Hence, *Tribolium* is likely to represent the ancestral state in insects.

In striking analogy to *Drosophila*, the expressions of vertebrate eye field patterning genes start out as one midline spanning domain (e.g. *Rx* and *Pax6* [65], [168], [169]). Later, these domains split medially, which is the prerequisite for the formation of bilateral eye anlagen. *shh* as well as *six3* are involved in medial repression of *Pax6* and Rx2 [65], [169] with *six3* acting upstream of *shh* [170]. This appears to be more similar to the derived *Drosophila* situation than to the ancestral split of head lobe anlagen (see above). However, the molecules involved in median split are different (*dpp* in *Drosophila* versus *six3* and *shh* in vertebrates) and we find involvement of *Tribolium six3* but not *dpp* in median patterning. Hence, the molecular data actually suggest a higher degree of conservation between *Tribolium* and vertebrates and convergent evolution of the similarity between *Drosophila* and vertebrates.

### Conserved Function of *six3* in Neuroendocrine and Anterior Median Patterning of Bilaterians

Regarding the likely difference to *Drosophila*, it is striking that the role of vertebrate *six3* in median separation of anterior expression domains is similar to what we find in *Tribolium*. In vertebrates, *six3* represses midbrain derived Wnt signaling [72], [73], which we also find in *Tribolium*. In vertebrates, *six3* and its paralog *six6* are involved in pituitary and hypothalamus development [76]–[80]. Based on its expression, *six3* has been predicted to contribute to neuroendocrine brain parts in annelids and *Drosophila* [81]. More generally, the similarity of bilaterian neuroendocrine systems and their common origin from placode like precursors have been noted [44], [47], [171]. Here, we have added functional data showing that *Tc-six3* is indeed required for the expression of neuroendocrine markers for the pars intercerebralis (*Tc-chx*) and pars lateralis (*Tc-fas2*) [141] placing it high in the hierarchy of neuroendocrine development in bilaterians.

In mouse embryos with reduced levels of *six3* and *shh* expression, median head and brain structures are affected (e.g. median nasal prominence) or absent (e.g. nasal septum, the septum, corpus callosum)(see Figure 5X) [170]. Such holoprosencephaly phenotypes are also seen in some human *six3* mutations [172]. Very similarly, we see loss of median brain structures in *Tribolium* after RNAi for *Tc-six3* Overall, these similarities functionally confirm that the ancestral role of *six3* orthologs was in the anterior median patterning of the Urbilateria (Figure 5V–5X) [81].

## Materials and Methods

### Animals

Most experiments were performed using the wild type *Tribolium castaneum* strain San Bernardino. For brain imaging, a transgenic line for 6XP3-ECFP (marking glia) and elongation factor1-alpha regulatory region-DsRedEx (EF1-B; marking neural cells) and the enhancer trap line Gö-11410 (marking mushroom bodies with EGFP; identified in the GEKU screen [173]) were used (Koniszewski, Kollmann, Averof, in preparation).

### Identification of Candidate Genes in *Tribolium*

Mouse protein sequences of the candidate genes (see Table S1) were obtained from the NCBI database (www.ncbi.nlm.nih.gov/). *Tribolium* orthologs were identified by BLAST at the Beetle Base server (beetlebase.org/). Test of orthology: *Tribolium* sequences were blasted against the NCBI protein database and the top 5–15 hits from insects, vertebrate and selected other groups were retrieved, as well as the three most similar *Tribolium* genes. These were BLASTed against the entire NCBI nucleotide database and the first three hits were retrieved. All these sequences were aligned using the ClustalW algorithm of Mega 4 [174],[175]. Phylogenetic trees were calculated in Mega 4 using the Neighbor-Joining method [176] (bootstrap consensus tree inferred from 10.000 replicates [177]; evolutionary distances computed using the Poisson correction method [178]; all positions containing gaps and missing data were eliminated from the dataset (complete deletion option)). See Figure S1 for phylogenetic trees. Phylogenetic relationships for the following genes were already published: *Tc-wnt11* and *Tc-wg/wnt1* [179], *Tc-otd1/otx* and *Tc-ems/emx* [54], *Tc-ey/pax6* and *Tc-toy/pax6* [136], *Tc-eya* [160].

### Cloning of Candidate Genes

mRNA of 0–48 h embryos was isolated using the MicroPoly(A)Purist Kit (Ambion) and cDNA was synthesized by using the SMART PCR cDNA Synthesis Kit (ClonTech). Gene fragments obtained by PCR with gene specific primers (see Table S3) were cloned into the pCRII vector using the TA Cloning Dual Promotor Kit (Invitrogen) and their sequence was confirmed.

### Whole-Mount In Situ Hybridization

Single (NBT/BCIP) and double in situ stainings (NBT/BCIP & FastRed or INT/BCIP) were performed and documented as described [180], [181].

### Knock Down of Gene Function by RNA Interference (RNAi)

RNAi was performed by injection of dsRNA into pupae (pRNAi), adults (aRNAi) or embryos (eRNAi) as described [182]–[184]. Lengths of gene fragments and mode of injection are listed in Table S1. Concentrations used in pupal RNAi and adult RNAi: 2–4 µg/µl; in embryonic RNAi: 1–2 µg/µl. A negative control for pupal RNAi was performed by pricking pupae with the needle or injecting water, injection buffer or dsRNA against tGFP. These controls did not show significant developmental effects in the offspring (not shown). In order to identify potential off-target regions, sequences were BLASTed against the *Tribolium* genome (BLASTn) at Beetle Base. For five genes, sequence similarity of 21 or more successive identical nucleotides was found to hit other gene predictions. For those, RNAi analysis was repeated by another person using subfragments that did not contain the potentially off-target sequences. The phenotypic effects were very similar with respect to the cuticle phenotype (not shown) and the head bristle pattern (Table S2). See Table S3 for primers for the subfragments. Because *Tc-six3* was investigated in more detail, two non-overlapping fragments were cloned, injected and analyzed separately by another person. Staining of *Tc-six3* in *Tc-six3* RNAi embryos confirmed strongly reduced or imperceptible expression in knock down embryos. The cuticle phenotype (not shown) and the head bristle pattern (Table S2) was very similar, confirming specificity.

### Epifluorescent and Confocal Imaging

Image stacks of cleared first instar cuticles were gathered by using a Zeiss LSM 510 or a Zeiss Axioplan 2 microscope and projections were calculated as described previously [44], [181]. Brain imaging was performed using a Zeiss LSM 510.

## Supporting Information

Figure S1 Phylogenetic analyses used to identify the orthologs of vertebrate neural plate patterning genes. A) Gene trees show orthology of the genes investigated here with vertebrate genes. Exceptions: Tc-Munster (mun) and Tc-Aristaless (al) are equally orthologous to Arx. However, *Tc-al* has been shown to be expressed in the distal appendages but not in the head and has therefore not been considered here [185]. Tc-Irx/Mirror and Tc-Iroquois (Tc-Iro) are equally orthologous to Irx. We find identical expression of both genes in the anterior neuroectoderm (not shown) and have worked with *Tc-irx*. B) Gene trees show orthology of the genes investigated here with vertebrate genes. C) Gene trees showing lack of orthologs for Atx, HesX1 and Vax genes in *Tribolium* and *Drosophila* genomes (Glean 05600 = Tc-Aristaless; Tc-simtoCG9930 = Tc-Ems; Is-Vax is the published name but our tree shows that it should be Is-Rough). Phylogenetic trees have already been constructed to determine the orthology of *Tc-ems* [54], *Tc-otd1* [130], *Tc-fgf8* [128], *Tc-wg* [179] and *Tc-ey/toy* [136]. Abbreviations of species: Vertebrates (Deuterostomia): *Hs: Homo sapiens; Mm: Mus musculus; Gg: Gallus gallus; Dr: Danio rerio; Xl: Xenopus laevis; Xt: Xenopus tropicalis; Rn: Rattus norvegicus*. Acorn worm (basal deuterostome): *Sk: Saccoglossus kowalevskii*. Tunicates: *Ci: Ciona intestinalis*. Annelid (Lophotrochozoa): *Pd: Platynereis dumerilii*. Crustacean (Ecdysozoa): *Ph: Parhyale hawaiiensis*. Insects (Ecdysozoa): *Dm: Drosophila melanogaster; Tc: Tribolium castaneum; Am: Apis mellifera; Ag: Anopheles gambiae; Bm: Bombyx mori; Aa: Aedes aegypti*. Chelicerates (Ecdysozoa): *Is: Ixodes scapularis*. Cnidarians (outgroup to Bilateria): Nv: Nematostella vectensis.
(PDF)

Figure S2 Genes with anterior and late expression. Gene expression depicted in blue with *Tc-wg* as red counterstain. Blastodermal stages are oriented with anterior to the left, germband stages are oriented with anterior to the top. The embryos in one row are staged according to their *Tc-wg* pattern and shape. Stages prior to onset of expression are omitted. (A) In addition to the *Tc-otd1* head domain (open arrowhead indicates anterior extreme of embryo), there is a midline expression domain (black arrowhead in A3–5). Early ubiquitous maternal expression is not shown [54], [130], [162]. (B) Expression of *Tc- six3* starts in the anterior-most part of the embryo in a ventral triangular domain. The region anterior to the *Tc-six3* domain is extraembryonic tissue (B1, ventral view). Later, *Tc-six3* marks anterior median tissue before it splits into a labral/stomodeal (black arrowheadin B3–4) and lateral domains (arrow in B3–4). De novo expression is found later in the eye anlagen (open arrowhead in B4–5) and in the mandibular segment (black stars in B5) [3], [81]. (C) *Tc-tll* starts expression at the posterior pole (arrow in C1) before the anterior domain covers the preocular lateral head lobes (C2–5) [131]. (D) *Tc-ptx* is expressed in bilateral spots in each segment (arrows in D1–2). Antennal and preocular domains are shown with open and black arrowhead, respectively [44].
(TIF)

Figure S3 Genes with anterior and segmental expression. Gene expression depicted in blue with *Tc-wg* as red counterstain except for A1 and B1 (*Tc-wg* in blue, *Tc-hh* in red) and B2–5 (no counterstain). Blastodermal stages are oriented with anterior to the left, germband stages are oriented with anterior to the top. The embryos in one row are staged according to their *Tc-wg* pattern and shape. Stages prior to onset of expression are omitted. (A) *Tc-hh* expression starts in anterior embryonic tissue of the blastoderm (open arrowhead in A1). In addition to the stripe posterior to the ocular *Tc-wg* domain, there is an anterior median domain (black arrowhead in A2). *Tc-hh* remains posterior to *Tc-wg* in the ocular segment (arrow in A3–4) and in all trunk segments and stomodeal expression arises (white asterisk in A3–5) [132]. (B) Also *Tc-wg* starts to be expressed at the blastoderm stage before marking the ocular region (open arrowheads in B2–5) and all other segments (mandibular segment marked with black arrowhead, antennal segment marked with arrow). In addition, stomodeal (white asterisk in B4) and labral expressions arises (grey arrow in B4–5) [134]. (C) *Tc-ci* expression starts in a broad domain in the entire pre-ocular region (open arrowheads in C1–4) and in the posterior portion of each segment (black stars in C2). In the early germband the median tissue is free of *Tc-ci* expression (arrow in C1) while during later stages labral domains arise (grey arrow in C3) [132]. (D) *Tc-irx* expression starts in an anterior median domain before retracting to an elongated posterior expression domain (arrows in D1–2). Strong expression around the stomodeum (white asterisk in D3), lateral to it (black arrowhead in D3–4) and median segmental expression arise later [44]. (E) *Tc-fgf8* marks large parts of the posterior blastoderm while in germbands it is most strongly expressed in median domains (black arrowhead in E2–5). In addition, strong expression is found in the antennal segment (arrow in E3) and in spots in the head lobes (white arrowhead in E4). (F) *Tc-slp* expression starts in anterior embryonic tissue (open arrowhead in F1) that develops into an ocular domain (open arrowheads in F2–5). Strong segmental expression arises (antennal segment marked by arrow). Eventually, additional domains in the labrum (grey arrow in F4–5) and in the lateral anterior head arise (black arrowhead in F4–5) [128]. (G) *Tc-ems* blastodermal expression (open arrowhead in G1) develops into a domain posterior to the ocular *Tc-wg* stripe (open arrowhead in G2) and later covers parts of the antennal segment (arrow in G3–4). Additional spots in the head lobes (black arrowhead in G5) and segmental expression arise subsequently [54]. (H) *Tc-ey* starts in the blastoderm and develops into a domain parallel to the ocular *Tc-wg* stripe (open arrowhead in H1–2) similar to *Tc-hh*, *Tc-wg*, *Tc-slp* and *Tc-ems*. This domain later covers part of the antennal and ocular segments (open arrowhead in H3–5) while additional paired domains arise in the neuroectoderm of each segment. The anteriormost of these domains contributes to mushroom body neuroblasts (black arrowhead in H4–5) [136].
(TIF)

Figure S4 Head bristle pattern marking the head capsule of *Tribolium* L1 larvae. After [54] Figure 1 but extended with dorsal ridge row.
(TIF)

Figure S5 Coexpression of *Tc-ey* with *Tc-dac* and coexpression of *Tc-six3* with neuroendocrine markers. (A–C) Coexpression of *Tc-ey* and *Tc-dac* mark putative mushroom body neuroblasts. The anterior median *Tc-ey* domain (white arrowhead in C) coexpresses *Tc-dac* (white arrowhead in C′ and C″). This is similar to the *Drosophila* situation where the combination of these transcription factors at a corresponding position identifies mushroom body neuroblasts. (D–G) Coexpression of *Tc-six3* (brown) and *Tc-chx* (blue) throughout development suggests that *Tc-six3* is expressed at the right place to act directly upstream of *Tc-chx* (D′–G′ are magnifications of the anterior median head region of the corresponding embryo). (H–K) Coexpression of *Tc-six3* (red) and anterior expression of *Tc-fas2* (blue) suggests that *Tc-six3* can act directly upstream of *Tc-fas2*. (L–O) *Tc-six3* RNAi embryos of different stages. The antennal, intercalary and mandibular *Tc-wg* stripes are marked with black arrow, black arrowhead and *md*, respectively in all panels. Despite the unusual orientation of the antennal *Tc-wg* stripes perpendicular to the body axis, they develop into regular antennae (seen in advanced embryonic stages and in cuticles).
(TIF)

Figure S6 Late aspects of expression of selected genes used for the fate map. Expression of *Tc-rx* (A), *Tc-irx* (B,C), *Tc-six3* (D,E), *Tc-chx* (E) and *Tc-labial* (F) in embryos at a stage when the head lobes are zippering together at the dorsal midline or are already dorsally fused (compare to schematic embryos in Figure 3F, 3I). (B,C) Black stars mark stomodeal expression and black arrowhead brain expression of *Tc-irx*.
(TIF)

Table S1 Orthologs, synonyms, and citations of studied genes.
(PDF)

Table S2 Quantification of head bristle pattern defects and off target controls.
(PDF)

Table S3 Genes with potential off target regions and primers used to clone subfragments without off target region.
(PDF)
