# Supplementary material for: Candidate Gene Screen in the Red Flour Beetle Tribolium Reveals Six3 as Ancient Regulator of Anterior Median Head and Central Complex Development
Source: PLoS Genet. 2011 Dec 22;7(12):e1002416. doi: 10.1371/journal.pgen.1002416 (PMC3245309; doi:10.1371/journal.pgen.1002416)

Fig. S1A: Phylogenetic trees of vertebrate neural plate patterning gene orthologs

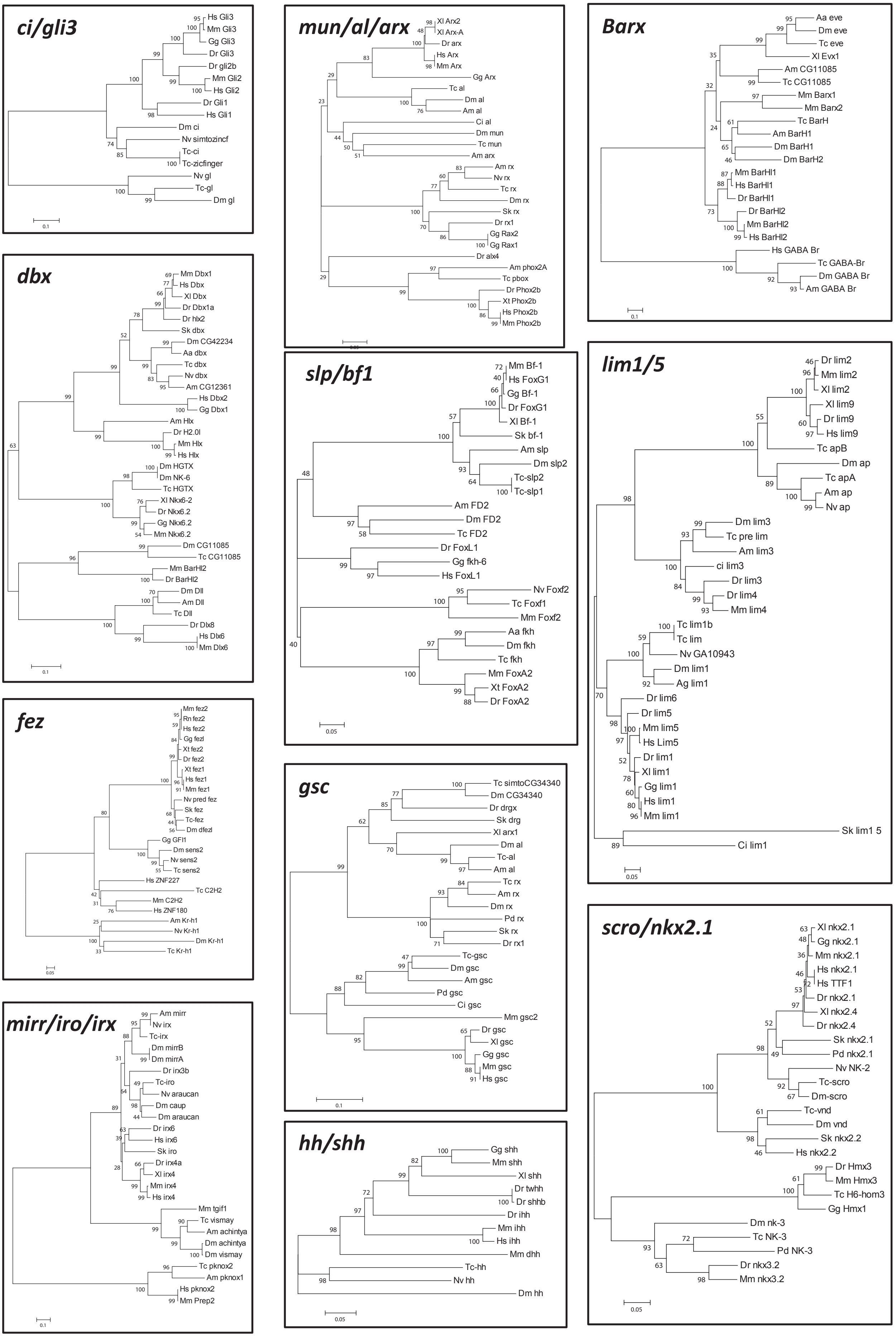

Fig. S1B: Phylogenetic trees of vertebrate neural plate patterning gene orthologs

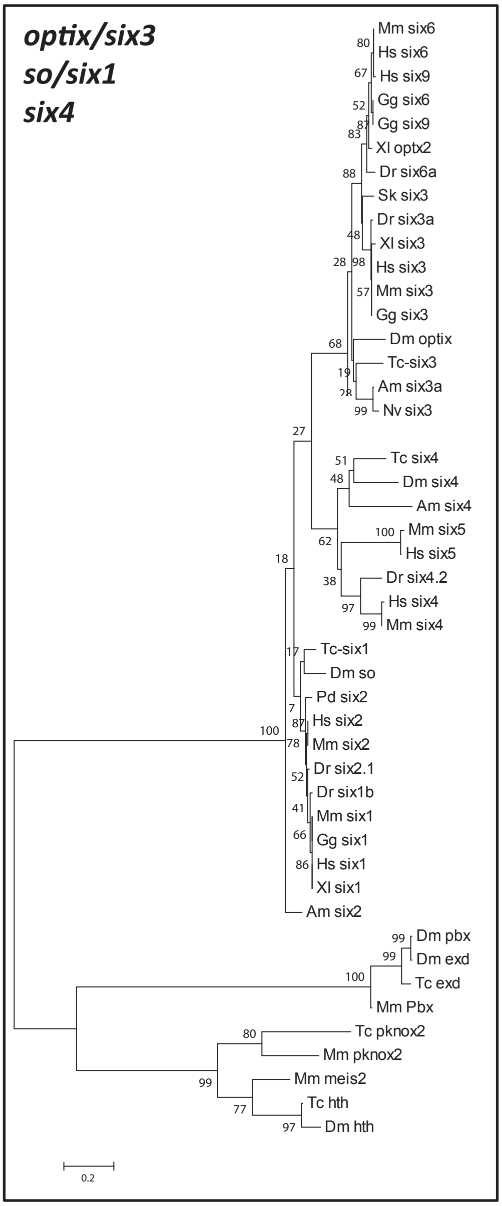

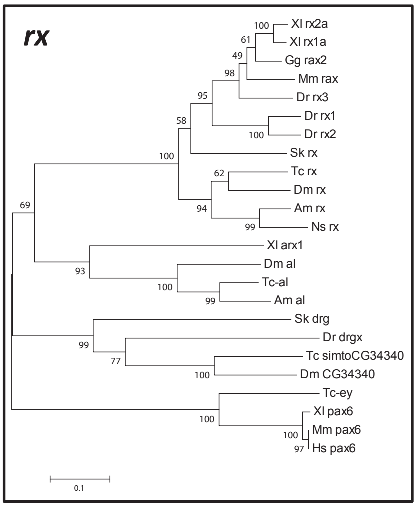

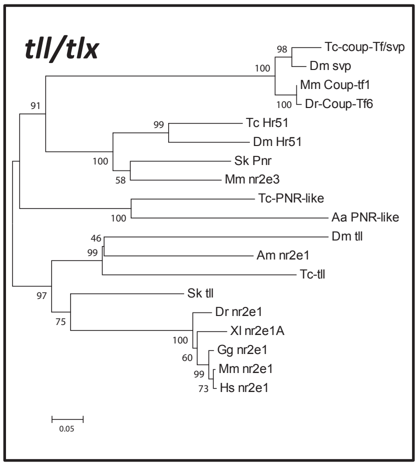

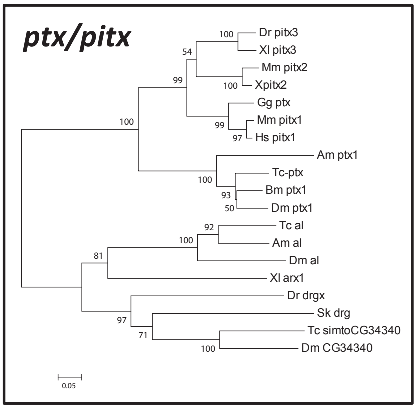

Fig. S1C: No orthologs in *Tribolium* and *Drosophila*

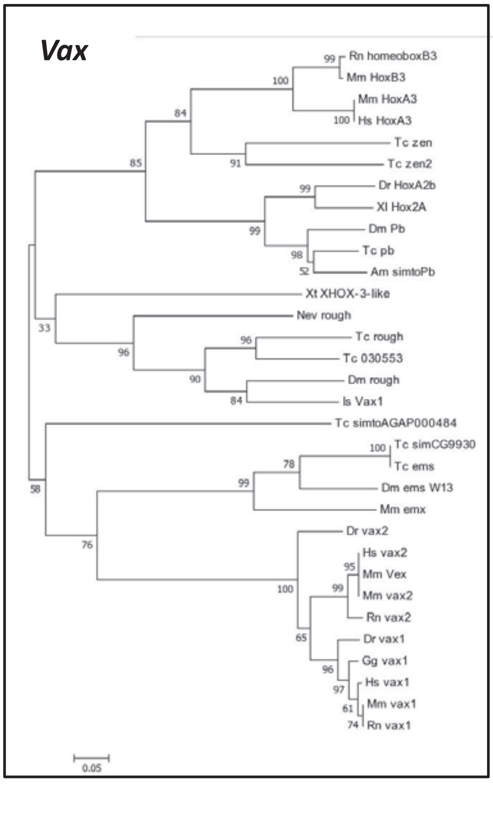

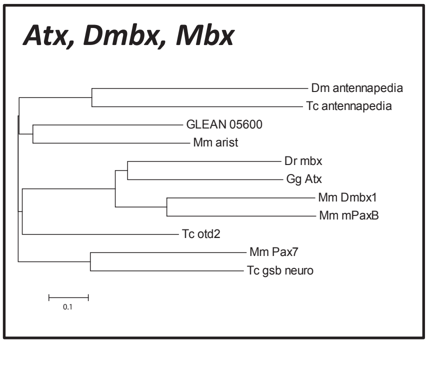

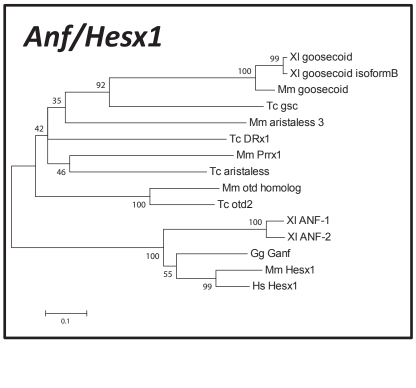

Supplement: Figure S1 — Phylogenetic analyses used to identify the orthologs of vertebrate neural plate patterning genes. A) Gene trees show orthology of the genes investigated here with vertebrate genes. Exceptions: Tc-Munster (mun) and Tc-Aristaless (al) are equally orthologous to Arx. However, Tc-al has been shown to be expressed in the distal appendages but not in the head and has therefore not been considered here [185]. Tc-Irx/Mirror and Tc-Iroquois (Tc-Iro) are equally orthologous to Irx. We find identical expression of both genes in the anterior neuroectoderm (not shown) and have worked with Tc-irx. B) Gene trees show orthology of the genes investigated here with vertebrate genes. C) Gene trees showing lack of orthologs for Atx, HesX1 and Vax genes in Tribolium and Drosophila genomes (Glean 05600 = Tc-Aristaless; Tc-simtoCG9930 = Tc-Ems; Is-Vax is the published name but our tree shows that it should be Is-Rough). Phylogenetic trees have already been constructed to determine the orthology of Tc-ems [54], Tc-otd1 [130], Tc-fgf8 [128], Tc-wg [179] and Tc-ey/toy [136]. Abbreviations of species: Vertebrates (Deuterostomia): Hs: Homo sapiens; Mm: Mus musculus; Gg: Gallus gallus; Dr: Danio rerio; Xl: Xenopus laevis; Xt: Xenopus tropicalis; Rn: Rattus norvegicus. Acorn worm (basal deuterostome): Sk: Saccoglossus kowalevskii. Tunicates: Ci: Ciona intestinalis. Annelid (Lophotrochozoa): Pd: Platynereis dumerilii. Crustacean (Ecdysozoa): Ph: Parhyale hawaiiensis. Insects (Ecdysozoa): Dm: Drosophila melanogaster; Tc: Tribolium castaneum; Am: Apis mellifera; Ag: Anopheles gambiae; Bm: Bombyx mori; Aa: Aedes aegypti. Chelicerates (Ecdysozoa): Is: Ixodes scapularis. Cnidarians (outgroup to Bilateria): Nv: Nematostella vectensis. (PDF) [file pgen.1002416.s001.pdf]
