# Supplementary figures and images for: Candidate Gene Screen in the Red Flour Beetle Tribolium Reveals Six3 as Ancient Regulator of Anterior Median Head and Central Complex Development
Source: PLoS Genet. 2011 Dec 22;7(12):e1002416. doi: 10.1371/journal.pgen.1002416 (PMC3245309; doi:10.1371/journal.pgen.1002416)

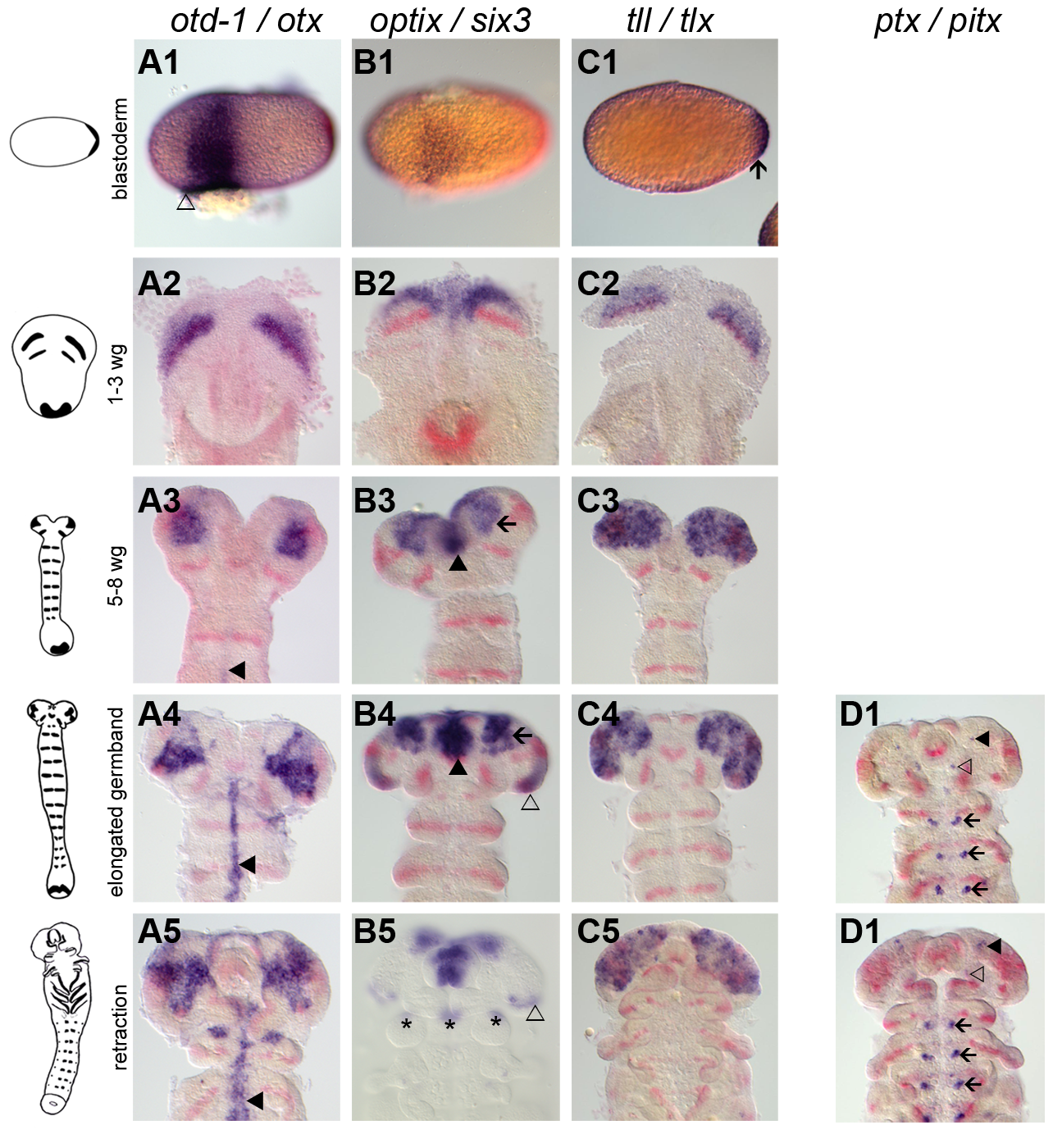

Supplement: Figure S2 — Genes with anterior and late expression. Gene expression depicted in blue with Tc-wg as red counterstain. Blastodermal stages are oriented with anterior to the left, germband stages are oriented with anterior to the top. The embryos in one row are staged according to their Tc-wg pattern and shape. Stages prior to onset of expression are omitted. (A) In addition to the Tc-otd1 head domain (open arrowhead indicates anterior extreme of embryo), there is a midline expression domain (black arrowhead in A3–5). Early ubiquitous maternal expression is not shown [54], [130], [162]. (B) Expression of Tc- six3 starts in the anterior-most part of the embryo in a ventral triangular domain. The region anterior to the Tc-six3 domain is extraembryonic tissue (B1, ventral view). Later, Tc-six3 marks anterior median tissue before it splits into a labral/stomodeal (black arrowheadin B3–4) and lateral domains (arrow in B3–4). De novo expression is found later in the eye anlagen (open arrowhead in B4–5) and in the mandibular segment (black stars in B5) [3], [81]. (C) Tc-tll starts expression at the posterior pole (arrow in C1) before the anterior domain covers the preocular lateral head lobes (C2–5) [131]. (D) Tc-ptx is expressed in bilateral spots in each segment (arrows in D1–2). Antennal and preocular domains are shown with open and black arrowhead, respectively [44]. (TIF) [file pgen.1002416.s002.tif]

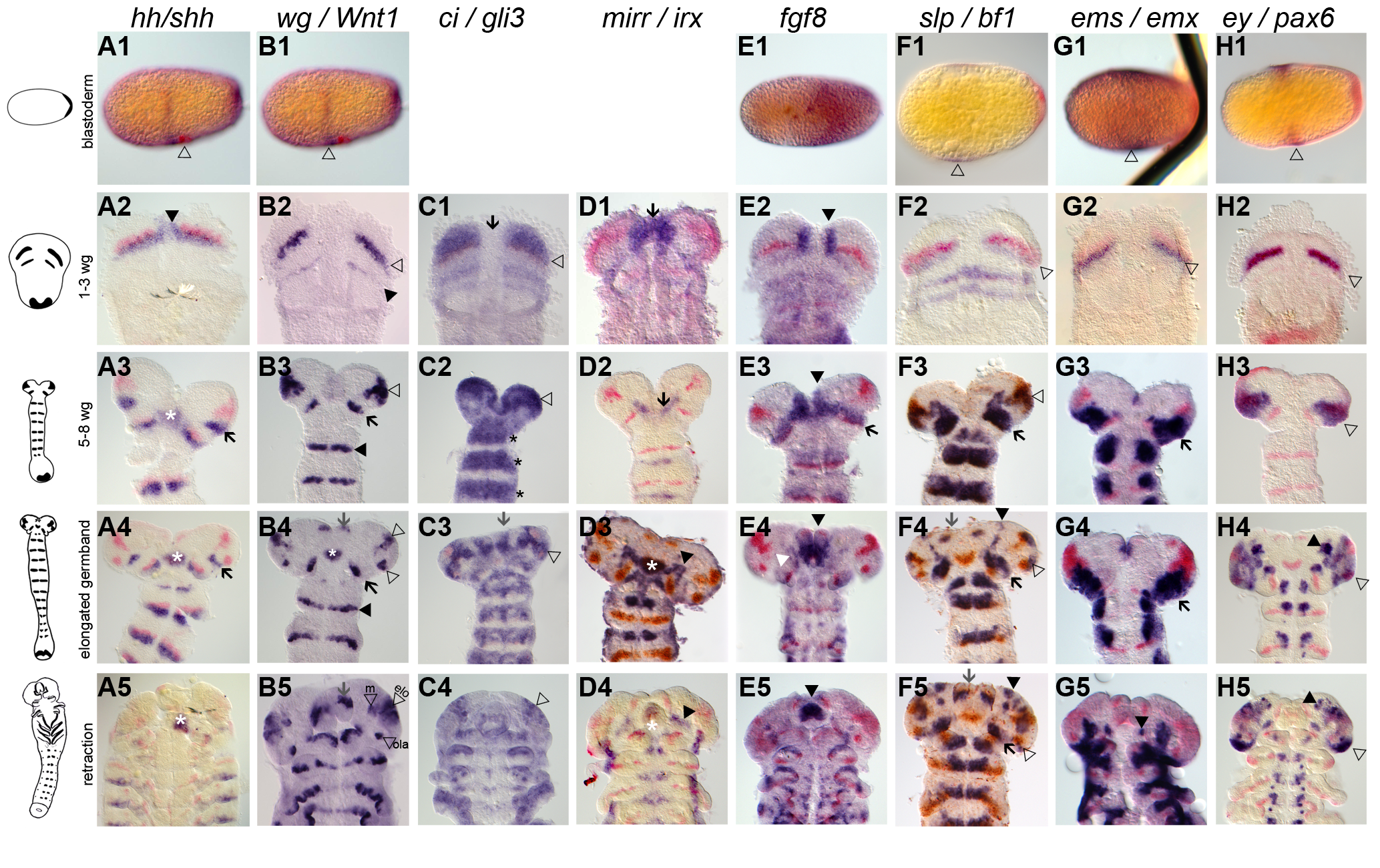

Supplement: Figure S3 — Genes with anterior and segmental expression. Gene expression depicted in blue with Tc-wg as red counterstain except for A1 and B1 (Tc-wg in blue, Tc-hh in red) and B2–5 (no counterstain). Blastodermal stages are oriented with anterior to the left, germband stages are oriented with anterior to the top. The embryos in one row are staged according to their Tc-wg pattern and shape. Stages prior to onset of expression are omitted. (A) Tc-hh expression starts in anterior embryonic tissue of the blastoderm (open arrowhead in A1). In addition to the stripe posterior to the ocular Tc-wg domain, there is an anterior median domain (black arrowhead in A2). Tc-hh remains posterior to Tc-wg in the ocular segment (arrow in A3–4) and in all trunk segments and stomodeal expression arises (white asterisk in A3–5) [132]. (B) Also Tc-wg starts to be expressed at the blastoderm stage before marking the ocular region (open arrowheads in B2–5) and all other segments (mandibular segment marked with black arrowhead, antennal segment marked with arrow). In addition, stomodeal (white asterisk in B4) and labral expressions arises (grey arrow in B4–5) [134]. (C) Tc-ci expression starts in a broad domain in the entire pre-ocular region (open arrowheads in C1–4) and in the posterior portion of each segment (black stars in C2). In the early germband the median tissue is free of Tc-ci expression (arrow in C1) while during later stages labral domains arise (grey arrow in C3) [132]. (D) Tc-irx expression starts in an anterior median domain before retracting to an elongated posterior expression domain (arrows in D1–2). Strong expression around the stomodeum (white asterisk in D3), lateral to it (black arrowhead in D3–4) and median segmental expression arise later [44]. (E) Tc-fgf8 marks large parts of the posterior blastoderm while in germbands it is most strongly expressed in median domains (black arrowhead in E2–5). In addition, strong expression is found in the antennal segment (arrow in E3) and i [file pgen.1002416.s003.tif]

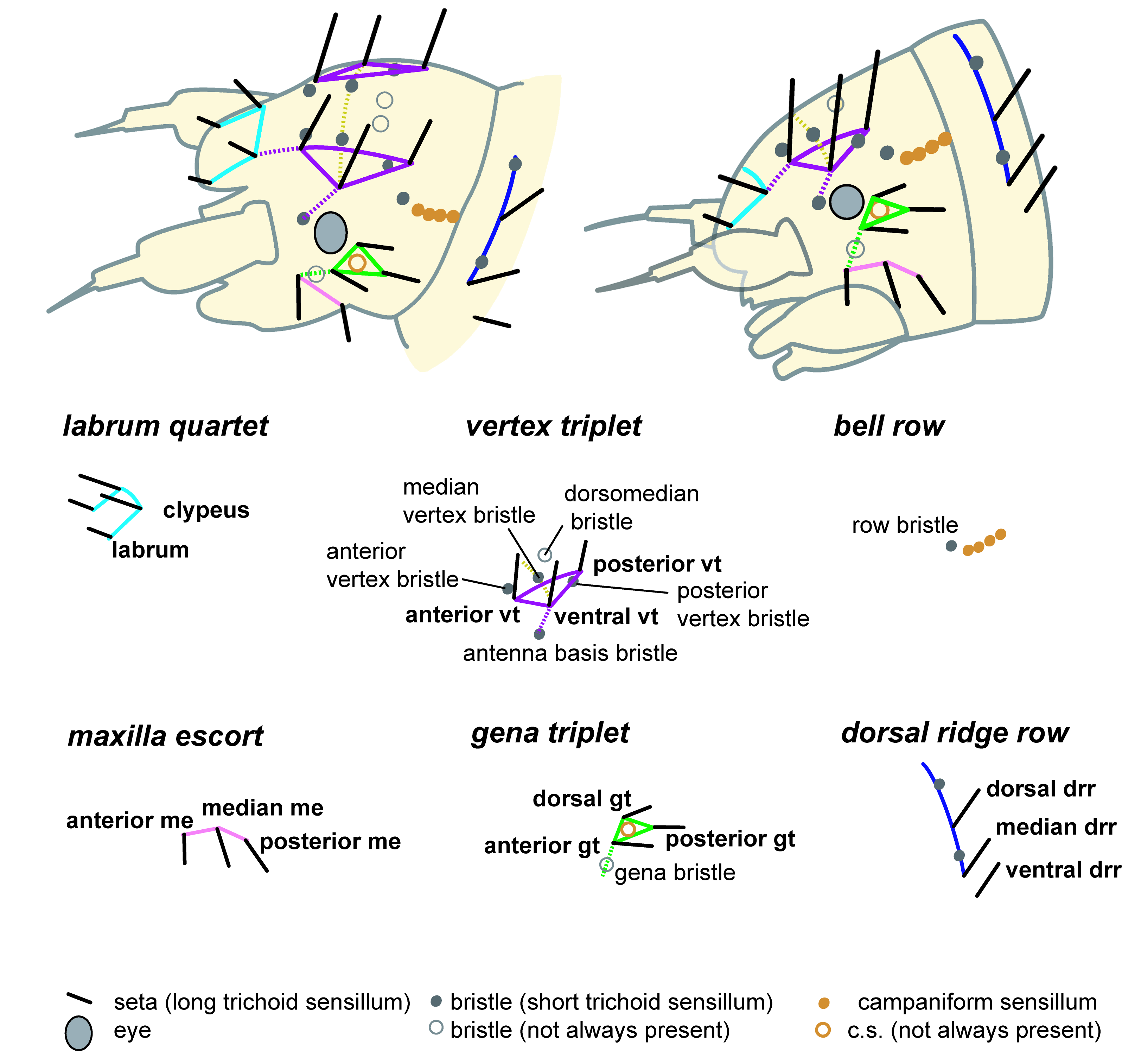

Supplement: Figure S4 — Head bristle pattern marking the head capsule of Tribolium L1 larvae. After [54] Figure 1 but extended with dorsal ridge row. (TIF) [file pgen.1002416.s004.tif]

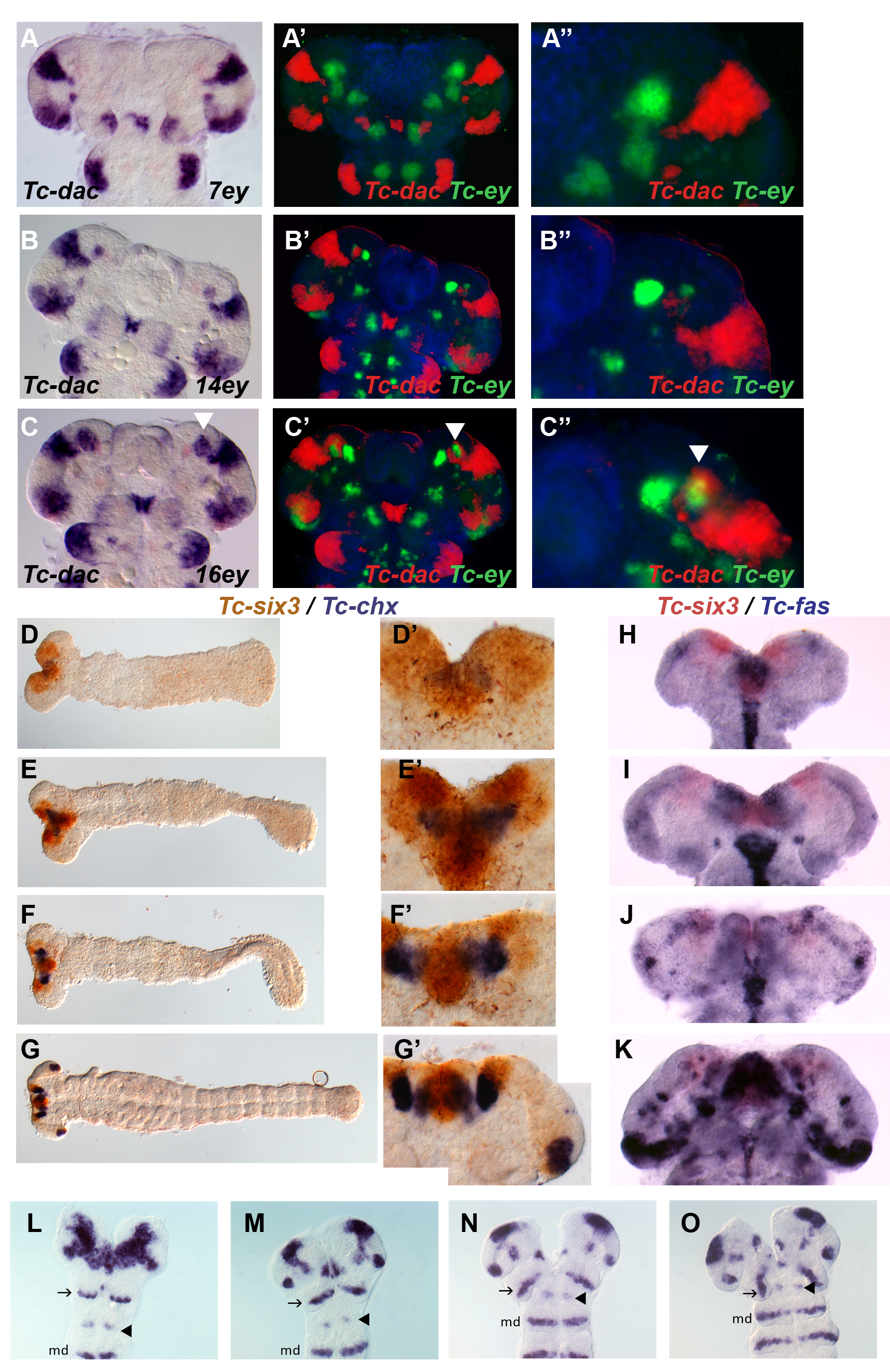

Supplement: Figure S5 — Coexpression of Tc-ey with Tc-dac and coexpression of Tc-six3 with neuroendocrine markers. (A–C) Coexpression of Tc-ey and Tc-dac mark putative mushroom body neuroblasts. The anterior median Tc-ey domain (white arrowhead in C) coexpresses Tc-dac (white arrowhead in C′ and C″). This is similar to the Drosophila situation where the combination of these transcription factors at a corresponding position identifies mushroom body neuroblasts. (D–G) Coexpression of Tc-six3 (brown) and Tc-chx (blue) throughout development suggests that Tc-six3 is expressed at the right place to act directly upstream of Tc-chx (D′–G′ are magnifications of the anterior median head region of the corresponding embryo). (H–K) Coexpression of Tc-six3 (red) and anterior expression of Tc-fas2 (blue) suggests that Tc-six3 can act directly upstream of Tc-fas2. (L–O) Tc-six3 RNAi embryos of different stages. The antennal, intercalary and mandibular Tc-wg stripes are marked with black arrow, black arrowhead and md, respectively in all panels. Despite the unusual orientation of the antennal Tc-wg stripes perpendicular to the body axis, they develop into regular antennae (seen in advanced embryonic stages and in cuticles). (TIF) [file pgen.1002416.s005.tif]

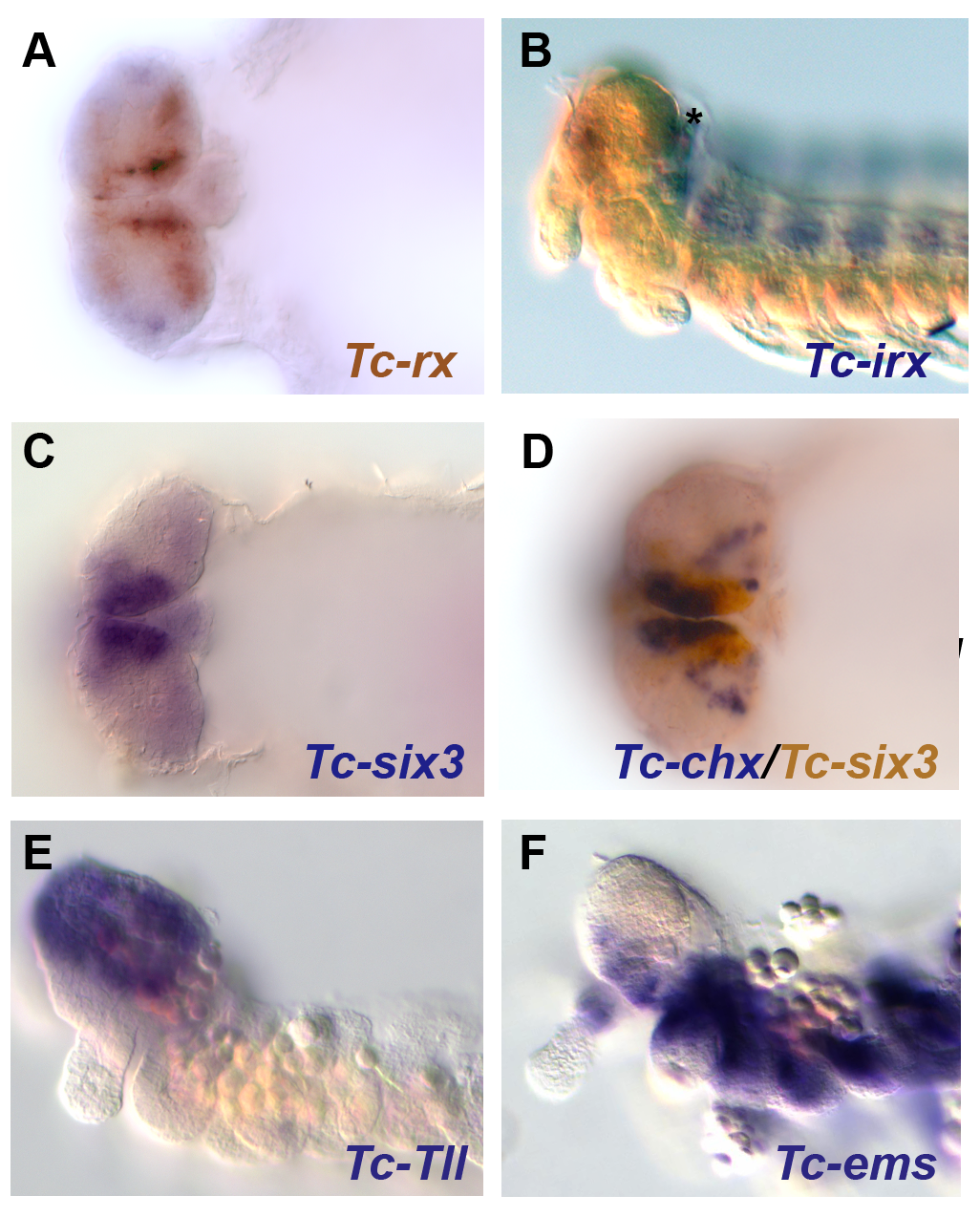

Supplement: Figure S6 — Late aspects of expression of selected genes used for the fate map. Expression of Tc-rx (A), Tc-irx (B,C), Tc-six3 (D,E), Tc-chx (E) and Tc-labial (F) in embryos at a stage when the head lobes are zippering together at the dorsal midline or are already dorsally fused (compare to schematic embryos in Figure 3F, 3I). (B,C) Black stars mark stomodeal expression and black arrowhead brain expression of Tc-irx. (TIF) [file pgen.1002416.s006.tif]
