# Supplementary material for: Candidate Gene Screen in the Red Flour Beetle Tribolium Reveals Six3 as Ancient Regulator of Anterior Median Head and Central Complex Development
Source: PLoS Genet. 2011 Dec 22;7(12):e1002416. doi: 10.1371/journal.pgen.1002416 (PMC3245309; doi:10.1371/journal.pgen.1002416)
Supplement: Table S1 — Orthologs, synonyms, and citations of studied genes. (PDF) [file pgen.1002416.s007.pdf]

	vertebrate gene	synonyms	Reference for expression domains	Drosophila ortholog	synonyms	Reference for Tribolium	RNAi e	RNAi p	RNAi a	Frag. size (bp)	Protein domains
	**expressed in the head of Tribolium**										
1	***otx***		Boncinelli et al., 1993; Rubenstein et al., 1998; Rubenstein and Shimamura, 1997	*orthodenticle / otd1*	*ocelliless (oc)*	Li et al. 1996, Schinko et al., 2008; Schroder, 2003				1116	homeobox
2	***six3/6*** *; sine oculis-related homeobox 3/6 homolog*		Loosli et al., 1998; Oliver et al., 1995; Takahashi and Osumi, 2008	*six3*	*optix*	Posnien et al., 2009; Steinmetz et al., 2010	X	X		839	homeobox, SIX-domain
3	***Tlx***	nuclear receptor subfamily 2, group E, member 1 (Nr2e1)	Arendt and Nubler-Jung, 1996; Hollemann et al., 1998; Kitambi and Hauptmann, 2007; Monaghan et al., 1995; Yu et al., 1994	*tailless / tll*		Schroder et al., 2000		X		1600	VitaminD receptor, Zinc finger nuclear hormone receptor-type, Steroid hormone receptor
4	***lhx1/5*** *; LIM homeobox protein 1/5*	Lim1/5	Sheng et al., 1997	*Lim1/5*				X		1009	LIM type zinc-finger, homeobox
5	***nkx2.1*** *; NK2 homeobox 1*	Nkx2-1, T/EBP, thyroid transcription factor-1, tinman, Titf1, Ttf-1	Rubenstein et al., 1998; Rubenstein and Shimamura, 1997	*scarecrow / scro*				X		837	homeobox
6	***gsc*** *; goosecoid*		Camus et al., 2000; Lemaire et al., 1997	*goosecoid / gsc*	*PvuII-PstI homology 25 (Pph25)*			X		703	homeobox
7	***rx*** *; retinal homeobox*	retina and anterior neural fold homeobox (rax),	Chuang et al., 1999; Mathers et al., 1997; Meijlink et al., 1999 Deschet et al., 1999	*retinal homeobox / rx*	*bk50, wombat (wom)*			X		395	homeobox, paired-like homeodomain
8	***fezf*** *; Fez family zinc finger*	forebrain embryonic zinc-finger	Hashimoto et al., 2000; Hirata et al., 2006; Hirata et al., 2004; Jeong et al., 2007; Matsuo-Takasaki et al., 2000	*fezf*				X		561	zinc-finger, Engrailed homology 1 (Eh1) repressor motif

9	***shh*** *; sonic hedgehog*	Hhg1, Hx, Hxl3	Rubenstein et al., 1998	*hedgehog / hh*		Farzana and Brown, 2008				1148	Peptidase C46, Hedgehog/intein hint domain, Hedgehog amino-terminal signaling region, Intein splicing site
10	***wnt1***	int-1	McMahon et al., 1992; Rubenstein et al., 1998	*wingless / wg*		Nagy and Carroll, 1994				1700	Secreted growth factor Wnt protein
11	***gli*** *; GLI-Krüppel family member*	brachyphalangy (bph)	Aoto et al., 2002; Hebert and Fishell, 2008	*cunitus interruptus / ci*		Farzana and Brown, 2008		X		1351	zinc-finger
12	***irx*** *; Iroquois related homeobox*		Cavodeassi et al., 2001; Glavic et al., 2002; Gomez-Skarmeta et al., 1998; Takahashi and Osumi, 2008	*mirror /mirr*					X	350	homeobox, Iroquois-class homeodomain
13	***emx*** *; empty spiracles homolog;*		Boncinelli et al., 1993; Rubenstein et al., 1998; Rubenstein and Shimamura, 1997	*empty spiracles / ems*	*antenna (ant)*	Schinko et al., 2008				888	homeobox, Helix-turn-helix motif
14	***foxg1*** *; forkhead box G1*	brain factor 1 (bf1, bf-1)	Hebert and Fishell, 2008; Rubenstein et al., 1998; Rubenstein and Shimamura, 1997	*sloppy paired / slp*	*foxg, FD6, FD7*	Choe and Brown, 2007; Choe and Brown, 2009; Choe et al., 2006		X		1142	fork head box, Winged helix repressor DNA-binding domain
15	***Pax6*** *; paired box gene 6*	Dey, small eye	Rubenstein and Shimamura, 1997; Scholpp et al., 2003; Shimamura and Rubenstein, 1997	*eyeless / ey & twin of eyeless / toy*		Yang et al., 2009a		X		860 (ey); 859 (toy)	homeobox, paired-like homeodomain, Winged helix repressor DNA-binding domain
16	***dbx1*** *; developing brain homeobox 1*	Mmox C	Fjose et al., 1994; Gershon et al., 2000; Lu et al., 1992; Lu et al., 1994; Shoji et al., 1996	*dbx*				X		489	homeobox, Helix-turn-helix motif
17	***pitx*** *; paired-like homeodomain transcription factor*	pituitary homeobox	Dickinson and Sive, 2007; Dutta et al., 2005; Meijlink et al., 1999; Schweickert et al., 2001; Zilinski et al., 2005	*ptx*				X		946	homeobox, paired-like homeodomain

18	***fgf8** ; fibroblast growth factor 8*	Aigf	Shimamura and Rubenstein, 1997; Crossley and Martin, 1995	*fgf8*		Beermann and Schröder, 2008		X		625	Interleukin 1/heparin-binding growth factor

not expressed in the head of Tribolium

19	***arx;** aristaless related homeobox*		Colombo et al., 2004; El-Hodiri et al., 2003; Friocourt et al., 2006; Seufert et al., 2005	*munster / mun*	*PvuII-PstI homology 13 (Pph13)*					827	homeobox, Paired-like homeodomain
20	***wnt11***		Cavodeassi et al., 2005	*wnt11*						961	Secreted growth factor Wnt protein
21	***Barx** ; BarH like homeobox*		Jones et al., 1997	*BarH*	*B-H*					731	homeobox, Helix-turn-helix motif

not in the Tribolium/Drosophila genome

22	***Hesx1***	HES-1, Rpx, ANF	Thomas and Beddington, 1996; Martynova et al., 2004								homeobox
23	***Vax1** ; ventral anterior homeobox containing gene 1*		Hallonet et al., 1998								homeobox, Helix-turn-helix motif
24	***Dmbx1** ; diencephalon/mesencephalon homeobox 1*	Atx, Cdmx, Mbx, Otx3	Broccoli et al., 2002; Gogoi et al., 2002; Kawahara et al., 2002								homeobox, Paired-like homeodomain

e: embryonic RNAi, p: pupal RNAi; a: adult RNAi
