# Supplementary material for: Candidate Gene Screen in the Red Flour Beetle Tribolium Reveals Six3 as Ancient Regulator of Anterior Median Head and Central Complex Development
Source: PLoS Genet. 2011 Dec 22;7(12):e1002416. doi: 10.1371/journal.pgen.1002416 (PMC3245309; doi:10.1371/journal.pgen.1002416)
Supplement: Table S2 — Quantification of head bristle pattern defects and off target controls. (PDF) [file pgen.1002416.s008.pdf]

Head bristle defects

Gene	Beetles x2	bell row		vertex triplet							labrum quartet		gena triplet				maxilla escort		
				vertex setae			vertex bristles												
		bells	bristle	post	vent	ant	triplet	ant bas	median	ant	clypeus	labrum	post	dors	ant	bristle	ant	med	post
ci detailed	20	0	1	0	12	0	7 + 5	2 + 6	4 + 2	2 + 1	1	0	2	2	2	7	0	0	4
ci	20	0	1	0	12	0	12	8	6	3	1	0	2	2	2	7	0	0	4
%		0	5	0	60	0	60	40	30	15	5	0	10	10	10	35	0	0	20
slp2 detailed	20	0	4	16	0	8	16	2	13	8	8	0	0	0	0	1 + 8	2 + 10	2 + 10	6 + 7
slp2	20	0	4	16	0	8	16	2	13	8	8	0	0	0	0	9	12	12	13
%		0	20	80	0	40	80	10	65	40	40	0	0	0	0	45	60	60	65
six3 detailed	16	2	2	11 + 2	8 + 1	2 + 11	7 + 2	7	4 + 1	14	16	16	0	1	0	5	0	5	0
six3	16	2	2	13	9	13	9	7	5	14	16	16	0	1	0	5	0	5	0
%		12,5	12,5	81,25	56,25	81,25	56,25	43,75	31,25	87,5	100	100	0	6,25	0	31,25	0	31,25	0
lim1/5 detailed	24	0	5	1	1	0	1	2	1 + 1	2	0	0	2	2	2	16	6 + 1	9	0
lim1/5	24	0	5	1	1	0	1	2	2	2	0	0	2	2	2	16	7	9	0
%		0	20,83	4,1667	4,1667	0	4,1667	8,3333	8,3333	8,33	0	0	8,3333	8,3333	8,33333	66,6667	29,167	37,5	0
scro detailed	28	0	0	0	0	1	2	0	0	6	0	0	2	2	2	2	0	0	0
scro	28	0	0	0	0	1	2	0	0	6	0	0	2	2	2	2	0	0	0
%		0	0	0	0	3,5714	7,1429	0	0	21,4	0	0	7,1429	7,1429	7,14286	7,14286	0	0	0
dbx detailed	22	0	0	0	1	0	0	2	0	0	0	0	7	7	7	6 + 7 + 1	0	3	0
dbx	22	0	0	0	1	0	0	2	0	0	0	0	7	7	7	14	0	3	0
%		0	0	0	4,5455	0	0	9,0909	0	0	0	0	31,818	31,818	31,8182	63,6364	0	13,636	0
ptx detailed	20	6	9	3 + 2	0	0	1	2	2	1	0	0	0	0	0	4	0	0	0
ptx	20	6	9	5	0	0	1	2	2	1	0	0	0	0	0	4	0	0	0
%		30	45	25	0	0	5	10	10	5	0	0	0	0	0	20	0	0	0
fez detailed	26	0	7	1	1	1 + 4	1	2	1 + 1	1	0	0	0	0	0	15	0	6	0
fez	26	0	7	1	1	5	1	2	2	1	0	0	0	0	0	15	0	6	0
%		0	26,92	3,8462	3,8462	19,231	3,8462	7,6923	7,6923	3,85	0	0	0	0	0	57,6923	0	23,077	0
tll detailed	20	2	4	0	0	0	0	1	0	0	0	0	3	3	2	2 + 3	2	2 + 2	2
tll	20	2	4	0	0	0	0	1	0	0	0	0	3	3	2	5	2	4	2
%		10	20	0	0	0	0	5	0	0	0	0	15	15	10	25	10	20	10

rx	16	0	0	0	0	0	0	5	0	0	10	0	0	0	0	5	2	4	0
%		0	0	0	0	0	0	31,25	0	0	62,5	0	0	0	0	31,25	12,5	25	0
gsc	4	0	0	0	0	0	0	0	0	0	0	0	0	0	0	2	0	1	0
%		0	0	0	0	0	0	0	0	0	0	0	0	0	0	50	0	25	0
irx aRNAi	18	14	15	6	0	0	6	1	1	1	0	0	0	1	0	2	0	1	0
%		77,8	83,33	33,333	0	0	33,333	5,5556	5,5556	5,56	0	0	0	5,5556	0	11,1111	0	5,5556	0
toy detailed	12	10	12	4	5	0	12	11	3	0	0	0	1	1 + 1	1	10	0	10	0
toy	12	10	12	4	5	0	12	11	3	0	0	0	1	2	1	10	0	10	0
%		83,3	100	33,333	41,667	0	100	91,667	25	0	0	0	8,3333	16,667	8,33333	83,3333	0	83,333	0
ey	10	2	1	0	1	0	0	0	1	0	0	0	0	2	0	4	1	2	2
%		20	10	0	10	0	0	0	10	0	0	0	0	20	0	40	10	20	20
toy/ey detailed	6	6	6	2 + 2	6	0	6	6	3	0	0	0	2	4	3	5	2	6	5
toy/ey	6	6	6	4	6	0	6	6	3	0	0	0	2	4	3	5	2	6	5
%		100	100	66,667	100	0	100	100	50	0	0	0	33,333	66,667	50	83,3333	33,333	100	83,33

Off-Target controls

Gene	Beetles x2	bell row		vertex triplet							labrum quartet		gena triplet				maxilla escort		
				vertex setae			vertex bristles												
		bells	bristle	post	vent	ant	triplet	ant bas	median	ant	clypeus	labrum	post	dors	ant	bristle	ant	med	post
ci detailed	10	1	2	0	4+4	0	5+2	2	4+2	2+2	0	0	0	2	4	7	2	2	1
ci 1)	10	1	2	0	8	0	7	2	6	4	0	0	0	2	4	7	2	2	1
%		10	20	0	80	0	70	20	60	40	0	0	0	20	40	70	20	20	10
dbx detailed	20	1	3	0	2	0	2	1+1	4	0	0	0	5	3	5	4+5	1	2	0
dbx	20	1	3	0	2	0	2	2	4	0	0	0	5	3	5	9	1	2	0
%		5	15	0	10	0	10	10	20	0	0	0	25	15	25	45	5	10	0
toy detailed	10	3+3	9	3	3+2	1+1	9	9	3	0	0	0	3	0	2	8	3	9	3
toy	10	6	9	3	5	2	9	9	3	0	0	0	3	0	2	8	3	9	3
%		60	90	30	50	20	90	90	30	0	0	0	30	0	20	80	30	90	30
scro	20	0	2	0	0	0	2	1+1	1+1	4	0	0	2+1	1	0	2	0	0	0
scro 2)	20	0	2	0	0	0	2	2	2	4	0	0	3	1	0	2	0	0	0
%		0	10	0	0	0	10	10	10	20	0	0	15	5	0	10	0	0	0
rx 3)	30	0	5	6	1	0	2	17	3	11	10	0	0	0	0	10	2	6	0
%		0	16,67	20	3,3333	0	6,6667	56,667	10	36,7	33,333	0	0	0	0	33,3333	6,6667	20	0

six3 1 detailed	12	**0**	**4**	**7+1**	**9**	**7+5**	**10**	**7**	**7+1**	**7+1**	**12**	**12**	**2**	**1**	**0**	**8**	**1**	**0**	**0**
six3 4)	12	0	4	8	9	12	10	7	8	8	12	12	2	1	0	8	1	0	0
%		0	33,33	66,667	75	100	83,333	58,333	66,667	66,7	100	100	16,667	8,3333	0	66,6667	8,3333	0	0
six3 2 detailed	12	**0**	**0**	**7+2**	**9**	**8+2**	**6+4**	**7+1**	**9**	**10**	**6+4**	**6+4**	**1**	**1**	**0**	**6**	**0**	**2**	**0**
six3 4)	12	0	0	9	9	10	10	8	9	10	10	10	1	1	0	6	0	2	0
%		0	0	75	75	83,333	83,333	66,667	75	83,3	83,333	83,333	8,3333	8,3333	0	50	0	16,667	0

1) gnathum and abdomen affected 2) Lr split 3) Lr reduced 4) Lr absent

Color code numbers

- loss of bristle
- additional bristles
- misplaced bristles

Color code background

- 0%-14% = no phenotype
- 15% - 24% = very likely a phenotype
- >25% = significant phenotype
