# Supplementary material for: Candidate Gene Screen in the Red Flour Beetle Tribolium Reveals Six3 as Ancient Regulator of Anterior Median Head and Central Complex Development
Source: PLoS Genet. 2011 Dec 22;7(12):e1002416. doi: 10.1371/journal.pgen.1002416 (PMC3245309; doi:10.1371/journal.pgen.1002416)
Supplement: Table S3 — Genes with potential off target regions and primers used to clone subfragments without off target region. (PDF) [file pgen.1002416.s009.pdf]

Gene	specific nt	off-target nt	specific nt/off-targets nt	off-target genes	forward	reverse	size
Tc-ci	1351	44	30,7	*Tc-KRAB box and zinc finger, C2H2 type domain containing protein*<br><br>*Mm-PR domain containing 9 (Prdm9)*	TAATACGACTCACTATAGGCGCCAGGTTCGTCTTTGAGATC	T7	922bp
Tc-toy	859	23	37,3	*Tc-pox meso*	TAATACGACTCACTATAGGGCAACAACGACAACATCCCCAG	SP6-T7	589bp
Tc-scro	837	48	17,4	*Tc-gbx2*<br>*Tc-similar to AGAP000484-PA*	TAATACGACTCACTATAGGGAGATGAATCAACGAGGCCAGATG	SP6-T7	609bp
Tc-rx	395	21	18,8	*Tc-ptx*	ATGGAATCGGACCGTTGTGAAGA	GAACCATACCTGTACTCTGACTTCCG	474bp
Tc-dbx	489	23	21,3	*Tc-similar to AGAP000484-PA*	TAATACGACTCACTATAGGAAGGAGAGGGAATTGTTGGCG	T7	231bp
Tc-tll	1600	22	72,7	no gene prediction			
